# The PI3K Pathway Balances Self-Renewal and Differentiation of Nephron Progenitor Cells through β-Catenin Signaling

**DOI:** 10.1016/j.stemcr.2015.01.021

**Published:** 2015-03-05

**Authors:** Nils Olof Lindström, Neil Oliver Carragher, Peter Hohenstein

**Affiliations:** 1 The Roslin Institute, University of Edinburgh, Easter Bush Campus, Midlothian EH25 9RG, UK; 2 Edinburgh Cancer Research Centre, MRC Institute of Genetics and Molecular Medicine, University of Edinburgh, Western General Hospital, Edinburgh EH4 2XR, UK; 3 MRC Human Genetics Unit, MRC Institute of Genetics and Molecular Medicine, University of Edinburgh, Western General Hospital, Edinburgh EH4 2XU, UK

## Abstract

Nephron progenitor cells differentiate to form nephrons during embryonic kidney development. In contrast, self-renewal maintains progenitor numbers and premature depletion leads to impaired kidney function. Here we analyze the PI3K pathway as a point of convergence for the multiple pathways that are known to control self-renewal in the kidney. We demonstrate that a reduction in PI3K signaling triggers premature differentiation of the progenitors and activates a differentiation program that precedes the mesenchymal-to-epithelial transition through ectopic activation of the β-catenin pathway. Therefore, the combined output of PI3K and other pathways fine-tunes the balance between self-renewal and differentiation in nephron progenitors.

## Highlights

• Nephron progenitor cells require PI3K signaling for self-renewal
• Reduced PI3K activity enhances β-catenin-induced differentiation
• Nephron progenitor cells can differentiate prior to completion of epithelialization

## Graphical Abstract

**Figure:**
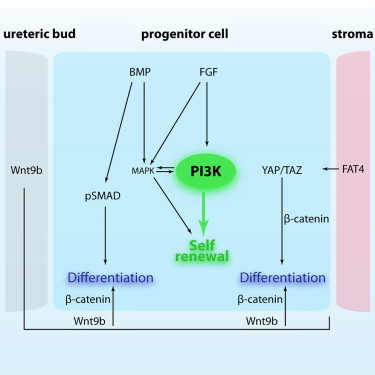

## Teaser

The fate of nephron progenitor cells is controlled by multiple signaling pathways. Hohenstein, Lindstrom, and colleague identify the PI3K pathway as a new player in this process. PI3K signaling affects the β-catenin-mediated control of these cells and has additional crosstalk with the BMP pathway. Moreover, PI3K inhibition shows differentiation and epithelialization of the nephron progenitor cells can be uncoupled.

## Introduction

Embryonic nephron progenitor cells (ENPs) form a population of cells that gives rise to all nephrons in a kidney (Costantini and Kopan, 2010). The balance between maintenance of ENPs and differentiation of the cells to form nephrons is essential for the development of a functional kidney. Multiple genes and signaling pathways have been shown to be involved in controlling this balance, including β-catenin signaling (Park et al., 2007), *Six2* (Self et al., 2006), *Fgf9/20* (Barak et al., 2012), *Bmp7* (Brown et al., 2013), *Osr1* (Xu et al., 2014), and *Fodxd1/Hippo/Yap* (Das et al., 2013). It may be surprising that mutations in so many, and maybe more, pathways all lead to a disturbance of this control. Two models for the control of ENPs can be envisioned. In one, each of these signals or pathways has its own discrete function, each of which is essential to control the balance between self-renewal and differentiation. The other possibility would be close crosstalk between signals, and convergence into one or a limited number of pathways that controls this balance. Close cooperation among the β-catenin, SIX2, and OSR1 proteins in the direct regulation of transcription of ENP genes has been demonstrated (Karner et al., 2011; Park et al., 2012; Xu et al., 2014), but the incorporation of other signals into a concise control mechanism remains to be demonstrated.

A potential point of convergence of multiple signals is the PI3K pathway, which acts downstream of receptor tyrosine kinases (RTKs) and G protein-coupled receptors and is negatively controlled by PTEN (Carracedo and Pandolfi, 2008). FGFs act directly on RTKs, YAP controls the expression of *miR-29*, which in turn targets PTEN, thereby increasing PI3K signaling (Tumaneng et al., 2012), and BMPs can control the PI3K pathway to regulate β-catenin-mediated signaling (He et al., 2004). FGFs and BMPs can both activate the MAPK pathway, which can be further modulated by PI3K signaling (Lanner and Rossant, 2010). Moreover, PI3K signaling has been shown to be important in controlling self-renewal of hematopoietic and embryonic stem cells (Paling et al., 2004; Perry et al., 2011).

Not much is known about PI3K signaling in the developing kidney. It was shown to be critical downstream of the GDNF/Ret system for normal branching morphogenesis (Kim and Dressler, 2007; Tang et al., 2002). In the nephrogenic lineage, overexpression of *Spry1*, an antagonist of RTKs, results in reduced expression of *Six2* and *Cited1*, two ENP markers, while in vitro recombinant FGF briefly can keep isolated ENPs in a CITED1^+^ state in a Ras/PI3K-dependent manner (Brown et al., 2011). We therefore set out to analyze the role of the PI3K pathway in the control of ENPs in more detail.

## Results

### ENP Maintenance Requires PI3K Signaling

**Figure 1:**
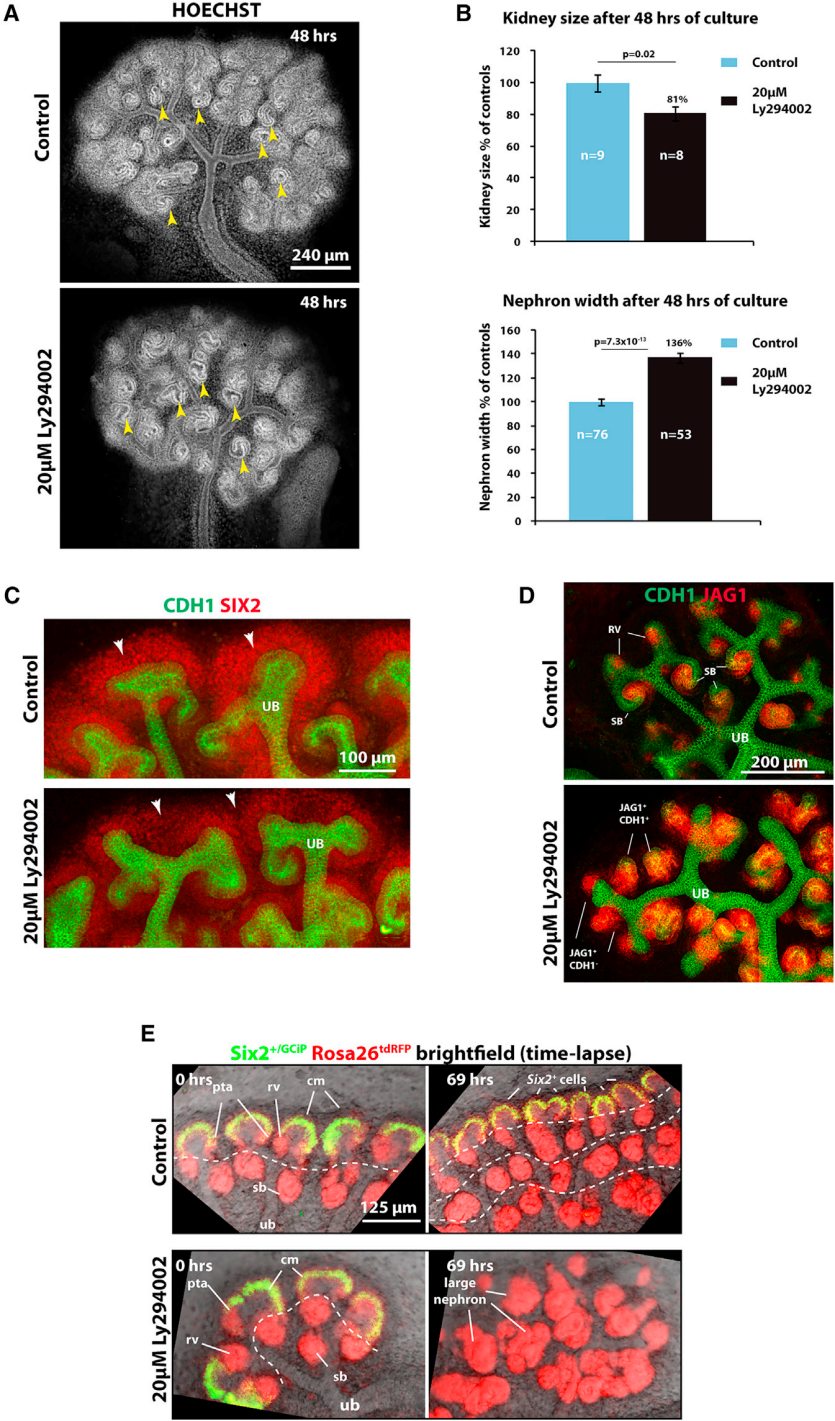
PI3K/Akt Signaling Is Necessary for ENP Self-Renewal and Kidney Development (A) E12.5 kidneys cultured for 48 hr. Arrowheads point to nephrons. (B) Measurements of kidney area and nephron tubule widths from kidneys. Nine and eight separate kidneys and 76 and 53 nephrons were analyzed for control and Ly294002 conditions, respectively. Error bars indicate SEM. Significance calculated using Student’s t test. (C and D) Kidneys cultured for 24 hr. Arrowheads indicate nephron progenitors. (E) Time-lapse data for E11.5 *Six2*^*+/GCiP*^*;Rosa26*^*tdRFP*^ kidneys cultured for 69 hr. White dashed line indicates generations of nephrons forming. Cm, cap mesenchyme containing ENPs; pta, pretubular aggregate; rv, renal vesicle; sb, s-shaped body; ub, ureteric bud; ubt, ureteric bud tip. Culture conditions and labeling are as indicated in figures. See also Figure S1.

To examine the role of PI3K during ENP self-renewal and differentiation, we blocked PI3K with LY294002 in kidney organ cultures. Treatment of kidney rudiments with this compound for 48 hr visually disturbed branching of the ureteric bud as described before (Tang et al., 2002; Figure 1A). PI3K inhibition resulted in reduced kidney size, but nephrons with increased diameter (Figure 1B) and a thinning of the SIX2^+^ ENP-containing cap mesenchyme (Figures 1A and 1C; Self et al., 2006) after blocking PI3K for just 24 hr, a time point when branching was still unaffected (not shown). We confirmed this phenotype using a second, structurally dissimilar PI3K inhibitor, GDC-0941 (Figures S1A–S1E; Table S1). Reduced staining for mTORC1 pSer2448 and AKT pSer473 confirmed the inhibition of the PI3K pathway by LY294002 (Figures S1F and S1G).

Ly294002 treatment of kidney rudiments for 24 hr resulted in the ectopic induction and formation of amorphous nephron structures (Figure 1D), suggesting an important role for PI3K signaling in ENP maintenance. We crossed *Six2*^+/GCiP^ mice expressing a CreGFP fusion from the endogenous *Six2* locus (Dolt et al., 2013) with *Rosa26*^+/tdRFP^ Cre reporter mice (Luche et al., 2007) to label ENPs GFP^+^/RFP^+^, whereas their post-mesenchymal-to-epithelial transition (MET) descendants would be GFP^−^/RFP^+^ as *Six2* would no longer be expressed. Time-lapse analysis of cultured embryonic kidneys showed that, whereas under control conditions *Six2* expression is maintained throughout the course of the experiment, PI3K inhibition leads to a rapid exhaustion of the ENPs as they differentiated into GFP^−^/RFP^+^ structures (Figure 1E; Figures S1H and S1I; Movie S1). Nephrons that had formed before Ly294002 treatment grew exceedingly large. After 96 hr, Ly294002 treatment had reduced both ureteric bud branching and nephron formation (Figure S1J). The average size of JAG1^+^ structures increased 6-fold. Although fewer nephrons formed, the total area of JAG1^+^ structures per kidney increased 2.7-fold. While PI3K inhibition increased apoptosis in the kidney, the apoptotic cells were mainly found surrounding the ENPs (Figure S1K), not in SIX2^+^ cells as found before (Motamedi et al., 2014). Combined, these data show that intact PI3K signaling is pivotal for the maintenance of ENPs.

### Differentiation and Epithelialization Can Be Uncoupled

**Figure 2:**
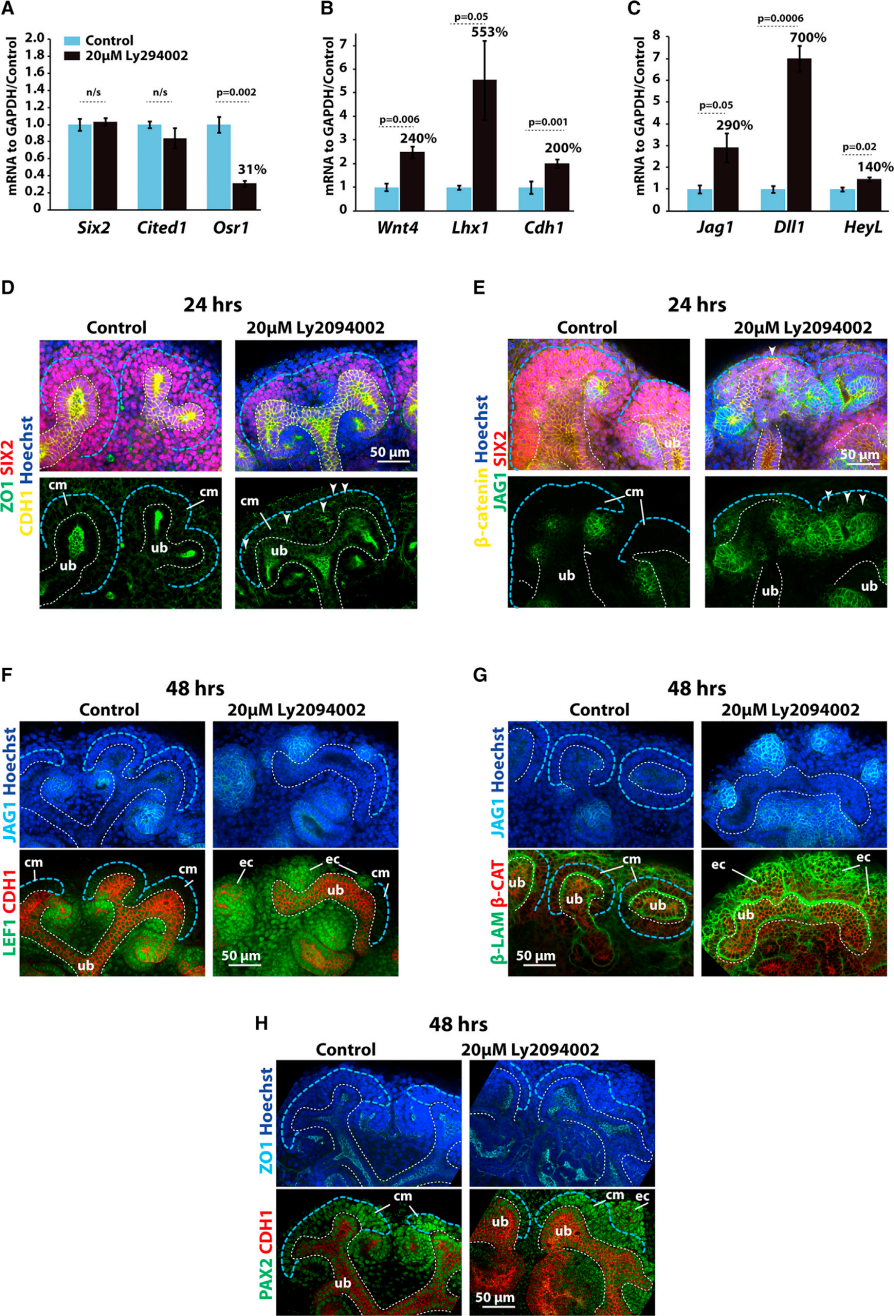
Nephron Progenitors Differentiate into Nephrons when PI3K Is Inhibited (A–C) qRT-PCR analyses on FACS-sorted cells from dissociated E12.5 *Six2*^*+/GCiP*^*;Rosa26*^*tdRFP*^ kidneys cultured for 24 hr. Cells from three kidneys were grouped to form each mRNA isolate replicate. Experiments were performed in triplicate with nine kidneys per treatment. All error bars indicate SEM. P values calculated using Student’s t test. (D and E) E12.5 kidneys cultured for 24 hr. (F–H) E12.5 kidneys cultured for 48 hr. Blue dashed line surrounds the nephron progenitor cells; white dashed line surrounds the ureteric bud; white arrowheads indicate points of ectopic expression. Cm, cap mesenchyme; ub, ureteric bud; ec, ectopic nephron. Culture conditions and labeling are as indicated in figures. See also Figure S2.

Current models for nephron development assume that mesenchymal ENPs undergo a MET before segment-specific expression programs are activated (Costantini and Kopan, 2010). We noted, however, that the ectopic nephrons that form under conditions of PI3K inhibition show signs of differentiation, for instance, expression of JAG1 and not all JAG1^+^ cells being fully epithelialized, as shown by the lack of CDH1 expression (Figure 1D). We analyzed this further at the 24 hr time point before the ENP population differentiated fully using qRT-PCR on RNA from *Six2*^+/GCiP^ *Rosa26*^+/tdRFP^ kidneys (Figures S2A–S2C). This confirmed that, in ENPs (GFP^+^/RFP^+^), cell expression of the ENP markers *Six2* and *Cited1* did not change after PI3K inhibition, though expression of *Osr1*, a marker of intermediate mesoderm that is maintained in the ENP stage, was reduced (Figure 2A). In contrast, in the same cells, Ly294002 treatment resulted in an upregulation of induction markers *Wnt4*, *Lhx1*, and *Cdh1* (Figure 2B) and segment markers *Jag1*, *Dll1*, and *HeyL* (Figure 2C). Note that, although we detected a modest upregulation of *Cdh1* mRNA 24 hr after PI3K inhibition (Figure 2B), at the same time point there was no sign of CDH1 protein expression in SIX2^+^ cells (Figure 1C). After 24 hr of PI3K inhibition, ectopic nephrons showed expression of JAG1 protein, while the tight junction marker ZO-1 and adherence junction protein β-catenin were increased in expression, but no CDH1 protein (MET marker, Figures 2D and 2E). After 48 hr in Ly2094002 expression of LEF1, PAX2 (induction markers), JAG1, and ZO-1 as well as CDH1 (Figures 2F–2H) confirmed that full MET eventually takes place in these structures. Expression of SIX2 was almost completely gone from cells that expressed CDH1 protein (data not shown).

### PI3K Signaling Modulates Endogenous β-Catenin Activity in ENPs

**Figure 3:**
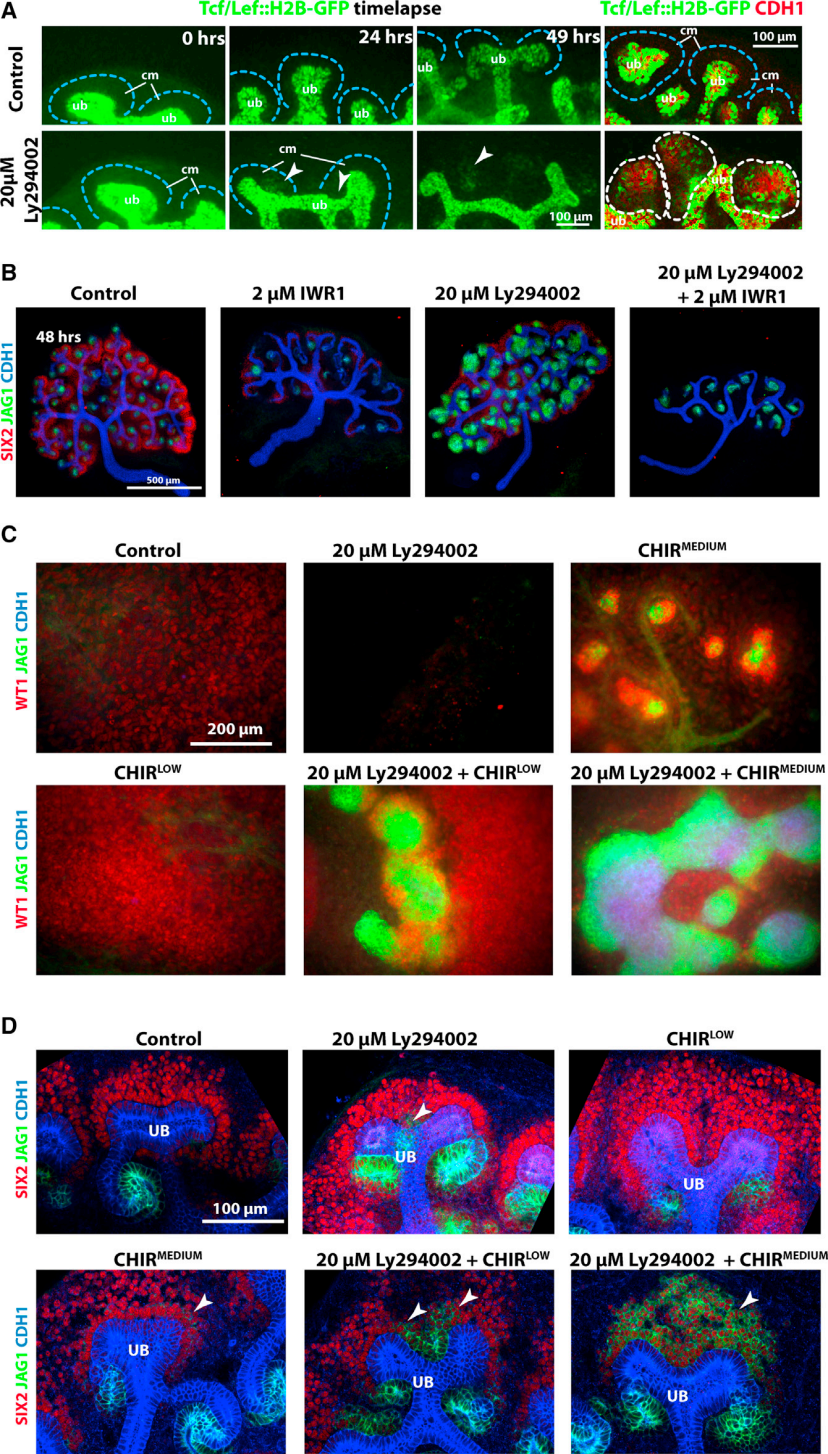
PI3K Inhibition Results in Ectopic Activation of β-cateninTcf/Lef Signaling in Nephron Progenitor Cells (A) Time-lapse data showing E12.5 *TCF/Lef::H2B-GFP* kidneys cultured for 49 hr. (Right) Fixed and stained kidneys. Blue dashed line surrounds the nephron progenitor cells; white arrowheads indicate points of ectopic sites of GFP expression; white dashed line outlines ureteric bud epithelium and indicates ectopic GFP expression also positive for Cdh1. (B) E12.5 kidneys cultured for 48 hr. (C) Isolated mesenchyme cultured without the ureteric bud. (D) Regions of ENPs and UBTs from whole kidneys cultured for 24 hr. Arrowheads indicate sites of ectopic expression. Cm, cap mesenchyme; ub, ureteric bud. Culture conditions and labeling are as indicated in figures. See also Figures S3 and S4.

The differentiation of ENPs is positively controlled by β-catenin activity in the ENPs in response to a WNT9B signal from the ureteric bud (Karner et al., 2011; Park et al., 2012). We used time-lapse analysis of the *TCF*/*Lef*::H2B-GFP β-catenin activity reporter mouse (Ferrer-Vaquer et al., 2010) to test the involvement of β-catenin in the ectopic nephrons obtained through PI3K inhibition. In control cultures, low-level activity of the reporter could be seen in the ENPs in the cap mesenchyme, and the signal increased in epithelialized CDH1^+^ nephrons (Figure 3A, top; Movie S2; Figures S3A and S3B). Ly294002 treatment of reporter kidneys resulted in activity of the β-catenin-signaling pathway in the ectopic nephrons, and these GFP^+^ structures later became CDH1^+^ (Figure 3A, bottom; Movie S2). To test if β-catenin activity is required for PI3K inhibition-induced nephron induction, we used IWR1, a Tankyrase inhibitor that results in inhibition of β-catenin signaling. We confirmed that IWR1 specifically reduced β-catenin targets and the β-catenin-signaling reporter in kidneys, and IWR1 effectively blocked Ly294002-induced ectopic nephrons (Figure 3B; Figures S3C–S3E).

To determine if PI3K inhibition is sufficient for ectopic nephron induction, we tried to induce nephron induction in isolated mesenchymes, where the WNT9B induction signal from the ureteric bud is absent, by inhibiting PI3K activity (Figure 3C). Neither control conditions nor treatment with Ly294002 showed any signs of nephron induction, as monitored by WT1, CDH1, and JAG1 immunostaining. This showed PI3K inhibition is not sufficient for nephron induction. To test if PI3K inhibition can have an additive effect on β-catenin signaling in the process, we used GSK3β inhibitor CHIR99021 to activate β-catenin signaling. It is known that high β-catenin activity can induce nephron formation, but inhibits the subsequent epithelialization (Davies and Garrod, 1995; Kuure et al., 2007; Park et al., 2007). We therefore titrated the dose of CHIR to find a concentration that allowed induction and differentiation CHIR^medium^ (1.5 μM), and one just insufficient to trigger induction and differentiation CHIR^low^ (0.75 μM). The combination of CHIR^low^ with Ly294002 resulted in robust nephron epithelialization and differentiation but CHIR^low^ treatment alone did not (Figure 3C), demonstrating an additive and potentially synergistic effect of inhibiting PI3K signaling on the β-catenin-mediated nephron induction process. Combining PI3K inhibition with CHIR^medium^ conditions led to massive epithelialization and differentiation far exceeding the effect of CHIR^medium^ alone, providing more evidence of this additive effect. We further confirmed this by staining for LEF1 and β-catenin protein, which were both upregulated by combined treatment with Ly294002 and CHIR, but not with Ly294002 on its own (Figure S3F). We also confirmed the synergistic effect with endogenous β-catenin signaling in intact kidneys with the ureteric bud still present (Figure 3D; Figure S4). After 24 hr of culture, we detected SIX2^+^/JAG1^+^ cells, and ENPs strongly expressing β-catenin, ZO1, LEF1, and β-laminin. Although we identified β-catenin^+^ and ZO1^+^ foci, we did not detect CDH1 in these cells.

### PI3K Signaling Interaction with Other Pathways

**Figure 4:**
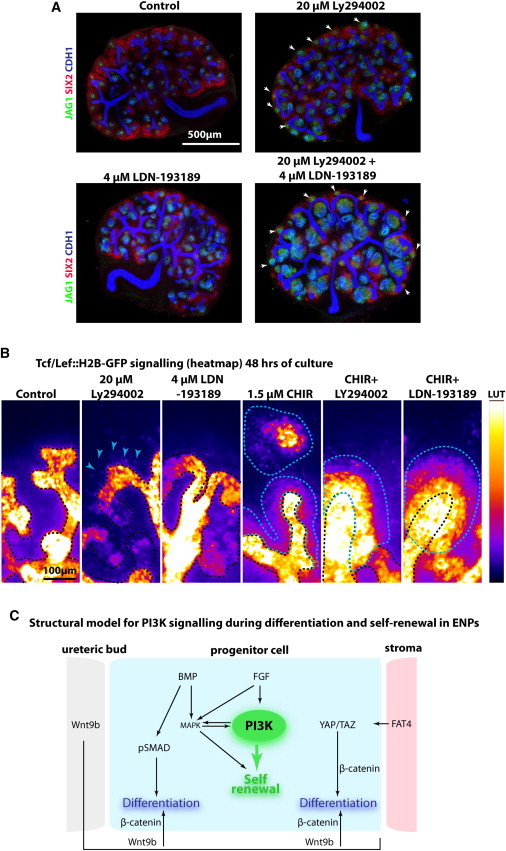
Multiple Signaling Pathways Feed into PI3K-Dependent ENP Self-Renewal (A) E12.5 kidneys cultured for 48 hr. White arrowheads indicate ectopic ENP differentiation. (B) Time-lapse data showing E12.5 *TCF/Lef::H2B-GFP* kidneys cultured for 48 hr. The GFP signal is shown as a heat map. Blue arrowheads indicate ectopic GFP^+^ nuclei; black dashed line outlines the ureteric bud and normally positioned nephrogenic epithelium; blue dashed line outlines ectopic regions with strong GFP signal. (C) Schematic model for PI3K signaling in ENP cells. The relationship of different signaling pathways is depicted and related to their outcomes in ENP cells. Culture conditions and labeling are as indicated in figures.

BMP7/pSMAD signaling can switch ENPs from self-renewing to differentiating (Brown et al., 2013). We compared the effects of inhibiting PI3K signaling to blocking BMP receptors with LDN-193189. Blocking BMP signaling led to a loss of SIX2^+^ cells, similar to that seen in *Bmp7*-deficient animals (Brown et al., 2013), and disruption of branching morphogenesis, but did not trigger ectopic nephron formation; inhibition of PI3K still drove ectopic nephron formation and altered the growth of nephrons when BMP signaling was inhibited (Figure 4A). Inhibition of BMP signaling did not trigger increased β-catenin signaling in ENPs (Figure 4B; Movie S3), but simultaneous inhibition of BMP signaling and activation of β-catenin actually resulted in massive and rapid upregulation of β-catenin signaling in ENPs. Inhibiting PI3K and activating β-catenin at the same time also resulted in massive upregulation of β-catenin signaling in ENPs, but the dynamics of the ENP response to CHIR + LDN-193189 and CHIR + Ly294002 were different. ENPs with only CHIR activated β-catenin signaling slower than those in CHIR + LDN-193189, and the cells were more motile and migrated away from the ureteric bud tips. In CHIR + LDN-193189, β-catenin signaling was activated very quickly, to higher levels, and the cells remained surrounding the ureteric bud tips. In CHIR + Ly294002 conditions, β-catenin signaling was activated quicker than in CHIR-only conditions, but, similar to CHIR + LDN-193189 conditions, however, cells displayed motility (Movie S3). Although CHIR + LDN-193189 and CHIR + Ly294002 created distinct responses from each other, they both increased the activation of β-catenin signaling compared to CHIR.

## Discussion

The data presented here support a role for PI3K signaling in controlling the balance between self-renewal and differentiation of ENPs. We showed that blocking the pathway with two structurally unrelated inhibitors leads to a rapid exhaustion of SIX2^+^ ENPs, as they differentiate and form large amorphous ectopic nephrons consisting of excessive numbers of differentiating ENPs. The cells differentiate directly where they are located and form nephron structures at positions around the whole of the ureteric bud tips (see lineage analyses of the ENPs in Movie S1). Our data show that PI3K function is coupled to the well-known role of β-catenin signaling in ENP balance control, but the differences we find between inhibiting PI3K and activating β-catenin show that PI3K signaling has additional β-catenin-independent roles as well. Our data on the combined inhibition of PI3K, BMPR, and GSK3β support a model where these pathways communicate with one another to regulate the stemness and differentiation of ENPs. The relative strengths of these pathways appear essential in this process, suggesting that self-renewal and differentiation are fine-tuned processes and not controlled by simple on/off control mechanisms.

A role for PI3K controlling ENPs fits well with current ideas of how FGF, BMP, β-catenin, and FAT4 regulate ENP self-renewal and differentiation (Figure 4C). The ENPs all receive the differentiation promoting β-catenin signal from the ureteric bud, but only the cells that receive a stromal signal differentiate (Das et al., 2013). The FAT4 signal from the stromal cells triggers phosphorylation and removal of YAP from the nucleus, thereby altering the transcriptional output of β-catenin (Das et al., 2013). As in other cell types, YAP signaling was shown to result in low levels of *miR-29*, increased PTEN, and inhibition of PI3K (Tumaneng et al., 2012). A model can be envisioned in which also this part of ENP control is fine-tuned by PI3K; however, this needs additional experimental verification.

In embryonic stem cells, PI3K inhibition has been shown to reduce β-catenin phosphorylation (Paling et al., 2004). In line with these findings, in our hands, inhibition of PI3K resulted in β-catenin signaling and simultaneous activation of β-catenin, and inhibition of PI3K had an additive effect on β-catenin activity. BMP signalling has previously been shown to be necessary for ENPs to respond to β-catenin activation in older kidneys, whereas cells from younger kidneys do not need this (Brown et al., 2013). In E12.5 kidneys, as used here, inhibition of BMP signaling did not on its own lead to ectopic nephron formation nor activation of β-catenin signaling. However, it was still possible to drive β-catenin signaling by inhibiting GSK3β, confirming that BMP signaling is not necessary for the ENPs to be able to signal via β-catenin during early kidney development. The dynamics of the ENP response to this dual inhibition/activation of BMPR and GSK3β was clearly different from that seen when β-catenin was activated on its own with the GSK3β inhibitor. This suggests that BMP signaling must still be controlling the induction process at this time point in kidney development, but perhaps via a different mechanism. Likewise, the dynamics of inhibiting PI3K signaling together with GSK3β was different from just altering GSK3β on its own or simultaneously blocking BMP and GSK3β. This again confirms that PI3K is not solely acting as a regulator of β-catenin signaling.

Although the different pathways that we investigated possibly converge to regulate PI3K signaling, they clearly also control separate processes. BMPR inhibition on its own did not trigger ectopic ENP differentiation, but when either BMPR or PI3K inhibitors were applied together with the GSK3β inhibitor, they both produced additive but still distinguishable effects. It recently has been shown that BMP and FGF signaling could be interacting in an antagonistic balance, where FGF promotes ENP survival and BMP/SMAD signaling controls apoptosis, and WT1 regulates both protein pathways (Motamedi et al., 2014). Untangling the very complex interactions among these pathways will require a significant effort in the future, and our study exemplifies the need to gently modify, rather than obliterate, signaling pathways, as can be achieved through careful use of inhibitors instead of full gene knockouts (Davies, 2009).

Unexpectedly, we found expression of nephron segmentation and epithelialization markers prior to the formation of CDH^+^ adherence junctions and the formation of a structurally distinct epithelium in PI3K-inhibited kidneys. We found that tight junctions began to form (ZO-1^+^) and β-catenin^+^ foci started to assemble, indicative of the formation of adherens junctions. Several other cadherins are expressed by the ENPs and could explain how β-catenin^+^ foci assembled without CDH1 (Goto et al., 1998; Klein et al., 1988). Further evidence of the ENPs beginning to epithelialize comes from the cells depositing a basement membrane (β-laminin^+^). Although, admittedly, inhibition of PI3K and simultaneous activation of β-catenin signaling does not necessarily reflect the normal situation, it does emphasize that shifts between the mesenchymal and epithelial states are fluid and dynamic transitions rather than sudden shifts. Indeed, while CDH1 expression can be detected only after the initial aggregation during nephrogenesis (Vestweber et al., 1985), proximal nephron marker and adhesion protein CDH6 can be detected in mesenchymal cells before CDH1 is detected (Cho et al., 1998). We have suggested previously that nephron segmentation starting before the formation of a rigid epithelium could explain the patterning defects in nephrons with reduced Rho-kinase activity (Lindström et al., 2013). ENPs can express genes associated with differentiation and segmentation prior to nephron formation (Brunskill et al., 2014). A better description of the dynamics of the renal MET is clearly necessary, and not just for semantic reasons. Understanding when markers are first expressed will help the phenotypic description of kidney development. Moreover, every step in the MET process is a potential moment when phenotypes can arise under experimental conditions, as shown here, or in disease situations.

## Experimental Procedures

Extended details outlining specific steps and protocols can be found in the Supplemental Experimental Procedures.

### Ethics Statement for Experimental Animals

All animal experiments were approved by the Edinburgh University Animal Welfare and Ethical Review Body, performed at the University of Edinburgh (UK), and carried out according to regulations specified by the Home Office and Project Licenses 60/3788 and 60/4473.

### Experimental Animals

For timed matings, noon of the day a vaginal plug was found was considered E0.5. CD1 animals were purchased from Charles River Laboratories. *TCF/Lef:H2B-EGFP* (Tg(TCF/Lef1-HIST1H2BB/EGFP)61Hadj) (Ferrer-Vaquer et al., 2010) were crossed with CD1s. *Six2*^+/GCiP^ (Dolt et al., 2013) mice were crossed with Rosa26^tdRFP^ (Gt(ROSA)26Sor^tm1Hjf^) (Luche et al., 2007).

### Organ Culture and Time Lapse

E12.5 kidneys were used. Kidney cultures were performed as described previously (Lindström et al., 2013). Isolated E11.5 mesenchyme was collected and induced as described previously (Davies and Garrod, 1995).

### Fluorescence-Activated Cell Sorting Analyses for Cell Analyses and RNA Isolation

*Six2*^+/GCiP^;*Rosa26t*^*dRFP*^ kidneys were dissociated into single cells and sorted for GFP and RFP using a FACSAriaIIIu (Becton Dickinson).

### RNA Analysis

RNA was isolated using RNeasy micro kits (QIAGEN). cDNA was generated using Superscript III and random primers. For the TaqMan reactions LightCycler 480 Probes Master (Roche) kits were used. Gene-specific primers and probes were designed using Ensemble IDs and the Roche Universal ProbeLibrary Assay Design Center. PCRs were multiplexed with *Gapdh* as an internal reference. Primer and probes are listed in the Supplemental Experimental Procedures. Pharmaceutical inhibitors are listed in Table S1. Inhibitors were used as specified in the text.

### Immunofluorescent Staining

Kidneys were fixed in −20°C methanol or in 4% paraformaldehyde (PFA) in 1×PBS for 20 min followed by −20°C methanol when fluorescent proteins were present. Primary and secondary antibody incubations were performed with antibodies diluted into 1×PBS at 4°C O/N. See list of antibodies and extended protocol in the Supplemental Experimental Procedures.

### Microscopy

Microscopy was performed on a Nikon TiE with 4×–10× objectives, or a Nikon A1R, N-STORM/A1, or a Zeiss LSM710 with 10×–63× objectives. Additional details can be found in the Supplemental Experimental Procedures. Quantitative image measurements are described in detail in the Supplemental Experimental Procedures.

## Author Contributions

N.O.L performed and analyzed all experiments. N.O.L. and P.H. designed the experiments. N.O.L prepared figures. N.C. advised on small molecule usage. N.O.L and P.H. wrote the manuscript.

## Supplemental Information

Document S1. Supplemental Experimental Procedures, Figures S1–S4, and Table S1

Movie S1. E11.5 *Six2CreGFP;tdRFP* Kidneys Cultured for 69 hr in Control Conditions or 20 ìM LY294002, Related to Figure 1 Time-lapse movie showing E11.5 *Six2CreGFP;tdRFP* kidneys cultured for 69 hr in the conditions specified in the movie. Images of bright field, GFP, and RFP channels were captured every 20 min. Times and scales are as indicated in the movie.

Movie S2. E12.5 *Tcf/Lef::H2B-GFP* Kidneys Cultured for 49 hr in Control Conditions or 20 ìM LY294002, Related to Figure 3 Time-lapse movie showing E12.5 *TCF/Lef::H2B-GFP* kidneys cultured for 49 hr in the conditions specified in the movie. Images of bright field and GFP channels were captured every 20 min. Red squares indicate magnified single-channel inserts, which show the growth of single ureteric bud tips (UBTs) and the surrounding nephron progenitor cells. Times and scales are as indicated in the movie.

Movie S3. E12.5 *Tcf/Lef::H2B-GFP* Kidneys Cultured for 48 hr in Specified Conditions, Related to Figure 4 Time-lapse movie showing six E12.5 *Six2CreGFP;tdRFP* kidneys cultured for 48 hr in the conditions specified in the movies. Images of bright field and GFP channels were captured every 20 min. Red square indicates magnified single-channel insert, which shows the growth of single UBTs and the surrounding nephron progenitor cells from each kidney. Times and scales are as indicated in the movie.

Document S2. Article plus Supplemental Information
