# Supplementary material for: The PI3K Pathway Balances Self-Renewal and Differentiation of Nephron Progenitor Cells through β-Catenin Signaling
Source: Stem Cell Reports. 2015 Mar 5;4(4):551–60. doi: 10.1016/j.stemcr.2015.01.021 (PMC4400645; doi:10.1016/j.stemcr.2015.01.021)
Supplement: Document S1. Supplemental Experimental Procedures, Figures S1–S4, and Table S1 [file mmc1.pdf]

**Stem Cell Reports, Volume *4***

**Supplemental Information**

# The PI3K Pathway Balances Self-Renewal and Differentiation of Nephron Progenitor Cells through β-Catenin Signaling

**Nils Olof Lindström, Neil Oliver Carragher, and Peter Hohenstein**

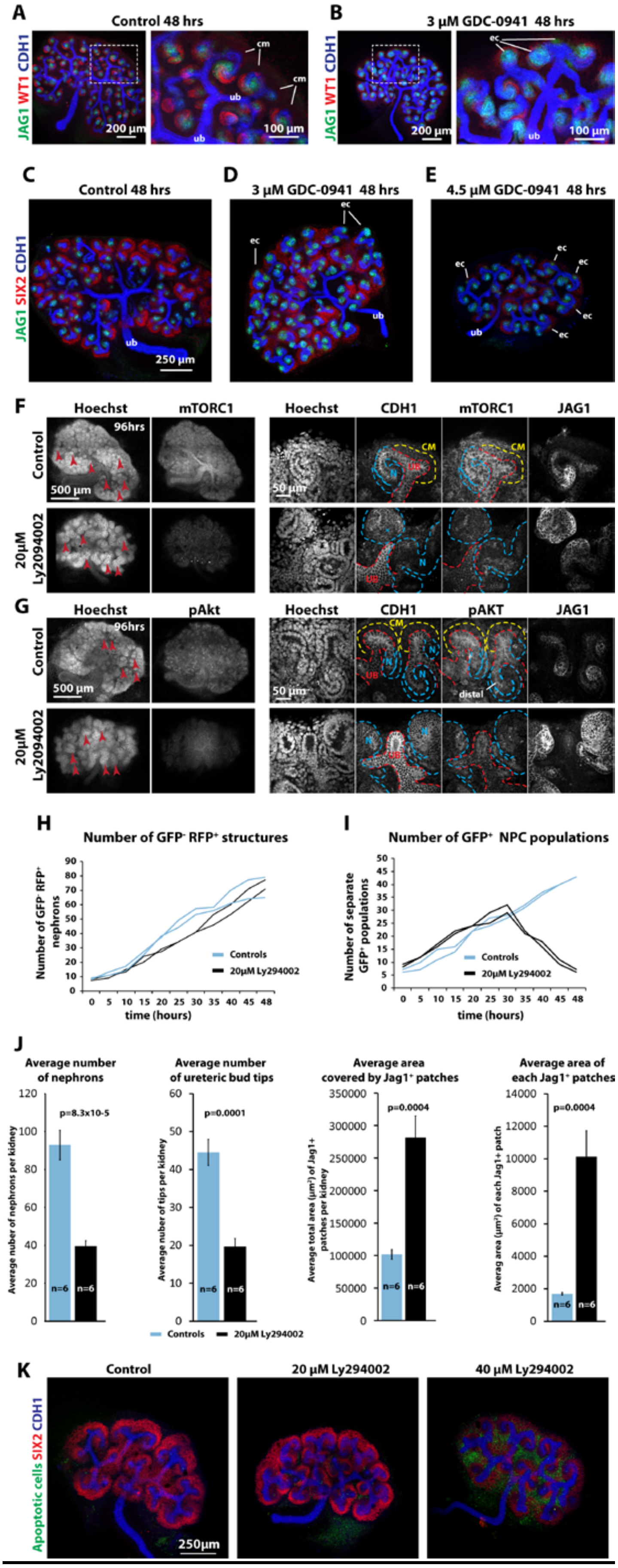
A
Control 48 hrs
B
3 µM GDC-0941 48 hrs
C
Control 48 hrs
D
3 µM GDC-0941 48 hrs
E
4.5 µM GDC-0941 48 hrs
F
G
H
Number of GFP- RFP+ structures
Controls
20µM Ly294002
time (hours)
I
Number of GFP+ NPC populations
J
Average number of nephrons
Average number of ureteric bud tips
Average area covered by Jag1+ patches
Average area of each Jag1+ patches
p=8.3x10-5
p=0.0001
p=0.0004
p=0.0004
K
Control
20 µM Ly294002
40 µM Ly294002
250µm

*FIGURE S2* *Lindström et al*

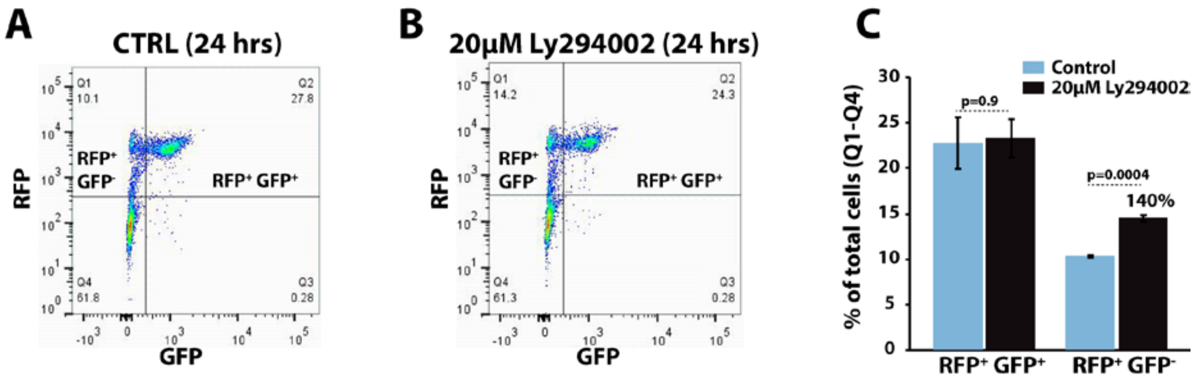

***FIGURE S3 Lindström et al***

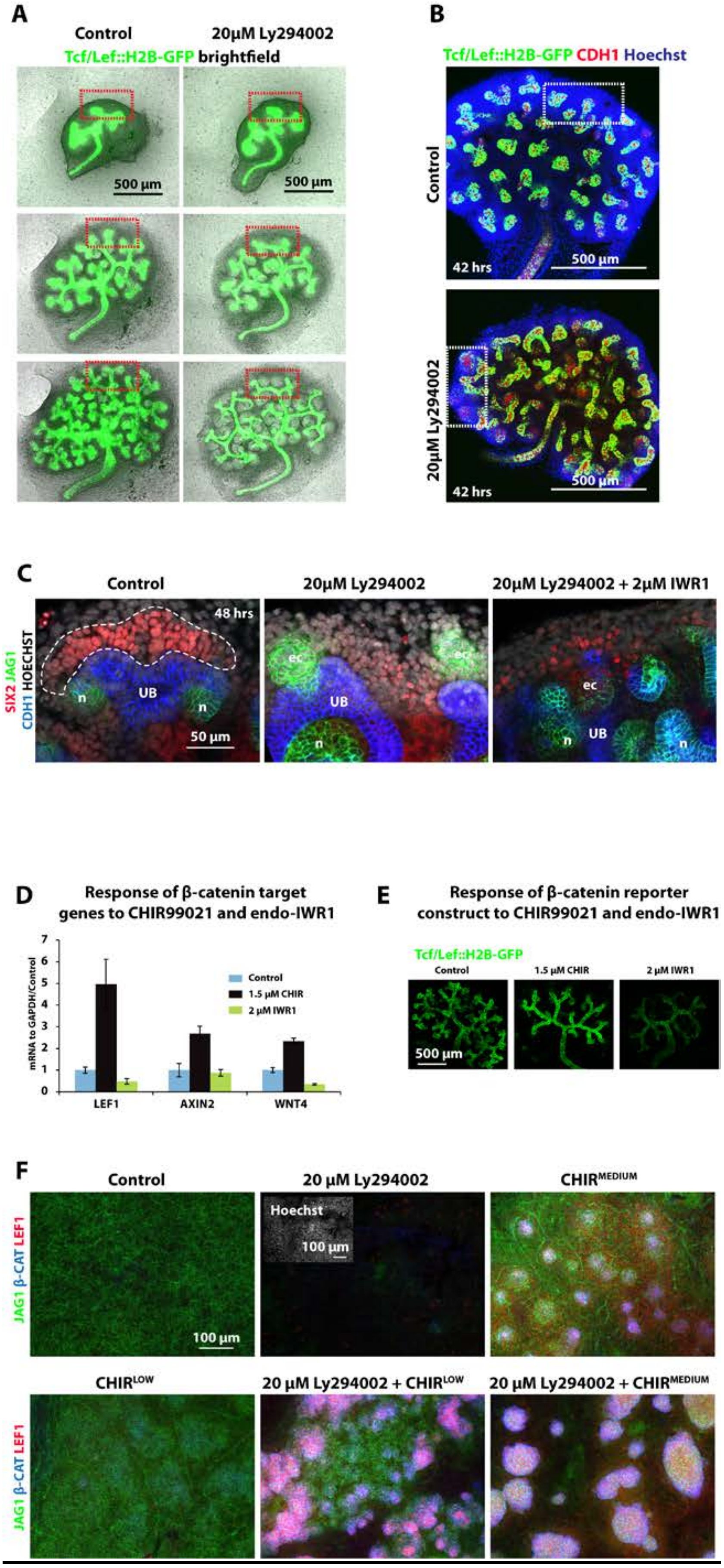

FIGURE S4 Lindström et al

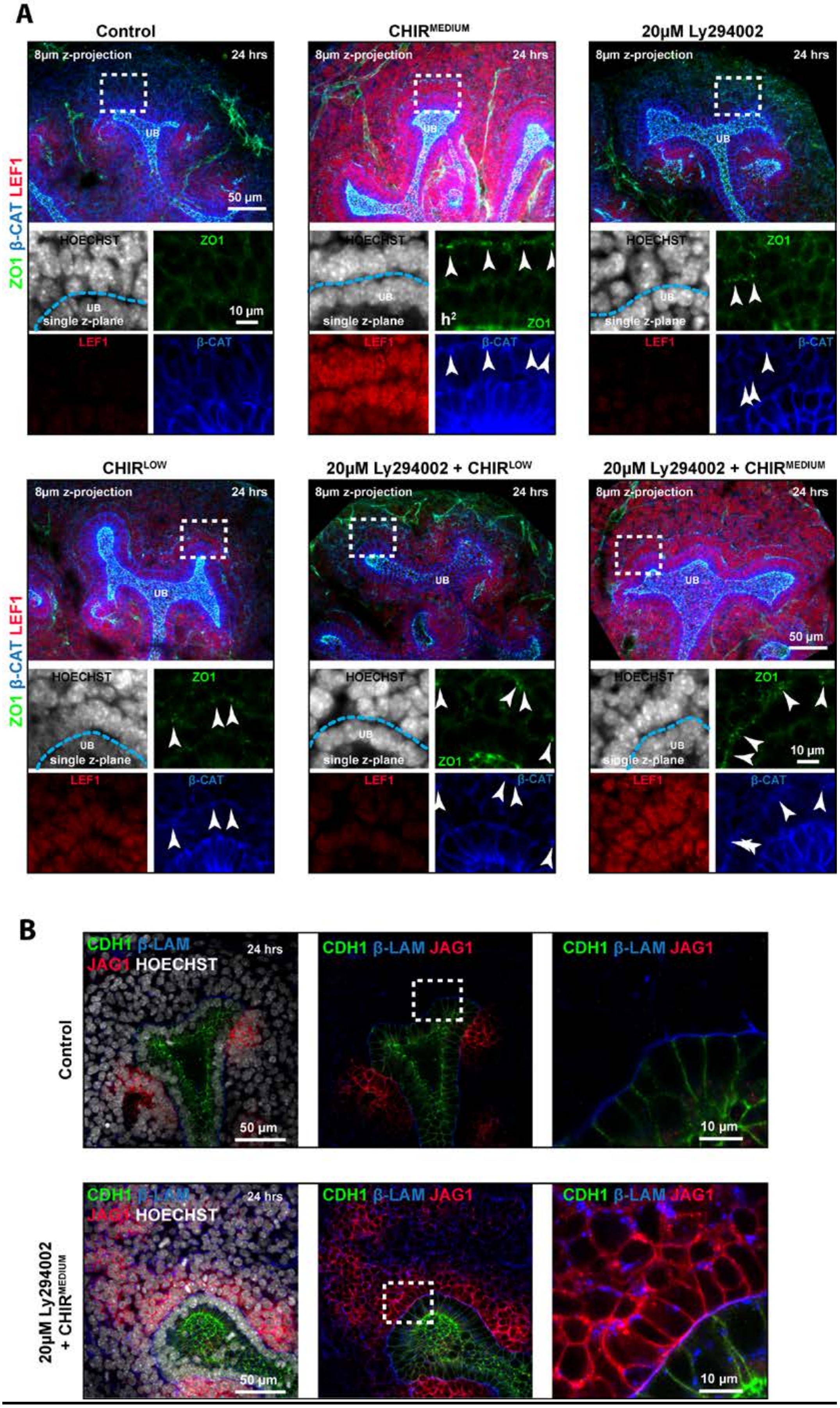

## Supplementary Figure legends

### Figure S1 – Relating to Figure 1. PI3K/Akt signalling is necessary for normal kidney development.

(**A-E**) Kidneys cultured for 48 hrs - square boxed indicate magnified areas shown. (**F-G**) E12.5 kidneys and magnified areas cultured for 96 hrs. Red arrowheads point to nephron tubules. Blue dashed lines indicate nephron structures. Red dashed lines indicate ureteric bud structures. Yellow dashed line indicates nephron progenitor cells. (**H-I**) Graphs showing time-lapse data from $Six2^{+/GCiP}$ *; Rosa26*$^{tdRFP}$ kidneys. (**H**) Graph showing the number of nephrons that formed over time. (**I**) Graph showing the number of separate nephron progenitor populations at each time-point. (**J**) Graphs showing average number of nephrons and ureteric bud tips per kidney, and average area of $JAG^{+}$ expressing patches and average area per kidney covered by $JAG1^{+}$ patches. Measurements made on kidneys after 96 hrs of culture. 6 kidneys were analysed per condition. Error bars indicate SEM. Significance calculated using Student's t-tests. (**K**) E12.5 kidneys cultured for 48 hrs. Ub- ureteric bud, ec-ectopic nephron. Culture conditions and labelling are as indicated in figures.

### Figure S2 – Relating to Figure 2. Isolation of ENPs from $Six2^{+/GCiP}$ *; Rosa26*$^{tdRFP}$ kidneys.

(**A-B**) FACS graphs of sorted E12.5 $Six2^{+/GCiP}$ *; Rosa26*$^{tdRFP}$. (**C**) Cell numbers as a percentage of the total. 9 kidneys were used per treatment. Error bars indicate SEM. Significance calculated using Student's t-tests.

### Figure S3 – Relating to Figure 3. PI3K inhibition results in ectopic activation of β-cateninTcf/Lef signalling in nephron progenitor cells.

(**A-B**) Time-lapse data and immunofluorescent stains showing E12.5 *TCF/Lef::H2B-GFP* kidneys cultured for 49 hrs. White and red squares indicate magnified areas displayed in (Figure 3A). (**C**) Regions of kidneys shown – relating to Figure 3B. (**D**) qRT-PCR analyses on mRNA isolated from whole E12.5 kidneys cultured for 48 hrs. mRNA isolated from triplicates of 3 kidneys with a total of 9 kidneys per condition. All error bars indicate SEM. (**E**) E12.5 *TCF/Lef::H2B-GFP* kidneys cultured for 48 hrs. (**F**) Isolated mesenchyme cultured

without the ureteric bud. Ub- ureteric bud, ec-ectopic nephron, n-nephron. Culture conditions and labelling are as indicated in figures.

**Figure S4 – Relating to Figure 3. Dual inhibition of PI3K and activation of β-cateninTcf/Lef signalling in nephron progenitor cells.**

(**A-B**) Immunofluorescent stains in regions of kidneys and ENPs from E12.5 kidneys cultured for 24 hrs. Culture conditions and labelling are as indicated in figures.

## Supplementary Movie legends

**Movie 1 – relating to Figure 1. E11.5 *Six2CreGFP;tdRFP* kidneys cultured for 69 hrs in control conditions or 20 μM LY294002.**

Time-lapse movie showing E11.5 *Six2CreGFP;tdRFP* kidneys cultured for 69 hrs in the conditions specified in the movie. Images of brightfield, GFP, and RFP channels were captured every 20 minutes. Times and scales are as indicated in the movie.

**Movie 2 – relating to Figure 3. E12.5 *Tcf/Lef::H2B-GFP* kidneys cultured for 49 hrs in control conditions or 20 μM LY294002.**

Time-lapse movie showing E12.5 *TCF/Lef::H2B-GFP* kidneys cultured for 49 hrs in the conditions specified in the movie. Images of bright-field and GFP channels were captured every 20 minutes. Red squares indicate magnified single-channel inserts which show the growth of single UBTs and the surrounding nephron progenitor cells. Times and scales are as indicated in the movie.

**Movie 3 – relating to Figure 4. E12.5 *Tcf/Lef::H2B-GFP* kidneys cultured for 48 hrs in specified conditions.**

Time-lapse movie showing 6 E12.5 *Six2CreGFP;tdRFP* kidneys cultured for 48 hrs in the conditions specified in the movies. Images of brightfield and GFP channels were captured every 20 minutes. Red square indicates magnified single-channel insert which shows the growth of single UBTs and the surrounding nephron progenitor cells from each kidney. Times and scales are as indicated in the movie.

**Supplemental Table**

**Table 1 – relating to all figures. Inhibitors, Concentrations, References**

| **Inhibitor** | **Target** | **Vehicle** | **Concentrations** | **Source** | **Cat. No.** | **Reference** |
|---|---|---|---|---|---|---|
| LY294002 | PI3K | DMSO | 20 - 40 µM | TOCRIS | 1130 | (Vlahos et al., 1994) |
| GDC-0941 | PI3K | DMSO | 3- 4.5 µM | Selleckchem | S1065 | (Folkes et al., 2008; Workman et al., 2010) |
| CHIR99021 | GSK3β | DMSO | 0.75- 1.5 µM | University of Dundee | - | (Meek et al., 2013; Ring et al., 2003) |
| LDN-193189 | BMPR | DMSO | 4 µM | STEMGENT | 04-0074 | (Cuny et al., 2008; Vogt et al., 2011) |
| endo-IWR1 | Tankyrase | DMSO | 2 µM | TOCRIS | 3532 | (Chen et al., 2009; Karner et al., 2010) |

**Supplemental Experimental Procedures with extended details**

**Organ culture and time-lapse** E12.5 kidneys were used throughout the work unless it was specified otherwise in the text. All organ cultures were performed at 37 °C with 5% $CO_2$ on 0.4 µm PET Transwell membranes (Corning). The standard culture media contained: DMEM (SIGMA), 10% FCS, and 1% Pen/Strep. For all data shown, a minimum of 3 kidneys were used to confirm general antibody stains in each condition and for samples where measurements were made at least 6 kidneys were used per condition, as specified in individual sections below.  Precautions were made to control for antibody variations, see section below: Quantitative image measurements.

To collect E11.5 mesenchyme, whole kidneys were gently trypsinized (2 min at RT), followed by trypsin neutralisation with full culture media. The mesenchyme was then peeled away from the ureteric bud and set up in a trans-filter set-up together with dorsal spinal-cord isolated from the same embryos as the kidneys were dissected from. This technique has previously been described in for example (Davies, 1994; Davies and Garrod, 1995). Three isolated mesenchymes were grouped and placed for each induction with spinal cord all inductions performed in triplicates.

**FACS analyses for cell analyses and RNA isolation** E12.5 *Six2*$^{+/GCiP}$; *Rosa26t*$^{dRFP}$ kidneys that had been cultured for 24 hrs were trypsinized (2 min at RT), neutralised in 150 µL 1xPBS with 10% FCS, left for 5 min, dissociated into single cells by pipetting, and passed through a cell-strainer. The cells were sorted for GFP and RFP using a FACSAriaIIIu (BD) into RLT buffer and used for mRNA isolation, cDNA synthesis, and gene expression analyses. Cells from 3 kidneys were grouped to form each mRNA isolate replicate. Experiments were performed in triplicate with 9 kidneys per treatment. P-values relating to changes in cell number due to treatments as stated on Graphs in Figure S2C. P-values relating to changes in gene expression as stated on Graphs in Figure 2A-C. Student's t-tests were used to compare conditions.

**Primary and Secondary Antibodies** Anti-CDH1 (BD Transduction Laboratories, 610182); Anti-JAG1 (R&D Systems, AF599), Anti-WT1 (Santa Cruz, sc-192), Anti-PAX2 (Covance, PRB-276P); Anti-SIX2 (LSBio, LS310189); Anti-phospho AKT (Cell Signalling, 4060); Anti-phospho mTORC1 (Cell Signalling, 5536); anti-LEF1 (Cell Signalling); Anti-β-catenin (BD Transduction Laboratories, 610154 ); Anti-ZO1 (DSHB R26.4C); Anti-β-laminin (SIGMA, L9393); Hoechst 33342 (Invitrogen, H3570).

Secondary antibodies against mouse, rabbit, goat, and rat IgG were purchased from Invitrogen. Anti-mouse IgG 488, A21202; Anti-rabbit IgG 594, A21207; Anti-goat IgG 488, A11055; Anti-goat IgG 594, A11058; Anti-mouse IgG 647, A31571; Anti-rabbit IgG 488, A21202; Anti-goat IgG 350, A21081; Anti-rabbit IgG 647, A31573; Anti-goat IgG 647, A21447.

NucView 488 Caspase-3 (Biotium, 30029-T-BT) was used to detect apoptosis in response to PI3K inhibition. Live cultures were incubated in 5 µM NucView for 60 min prior to fixation in 4% PFA and immunostaining for additional markers.

**Microscopy** The time-lapse microscopy was performed on a Nikon TiE (Perfect Focus System) with NIS-Elements 4.0. The imaging was performed using 4X or 10X objectives and a CoolSnap HQ2 CCD camera (Photometrics). The CoolSnap HQ2 CCD camera was used at a range of 0-16383. The microscope stage was enclosed within a humidified chamber at 37 °C with 5% $CO_2$. Images were captured every 20 minutes of relevant channels (brightfield/RFP/GFP). A combined exposure time for all channels was limited to 2s to prevent phototoxicity.

Confocal microscopy was carried out on a Nikon A1R using 10X-63X objectives, or a N-STORM/A1 super resolution microscope using 10X-63X objectives, or on a Zeiss LSM710 with 10X-63X objectives. Image stitching was carried out at 10X-20X to capture whole kidneys when required. The Nikon microscopes were used with Nikon NIS-Elements 4.0 and the Zeiss microscope with ZEN Black 2012. For image processing Fiji (http://fiji.sc/), ImageJ (http://rsb.info.nih.gov/ij/), and Adobe Photosphop CS5 were used. For image analysis Fiji and ImageJ were used. Time-lapse movies were presented using ImageJ and Fiji. Movies were generated in ZEN Blue 2012. Brightness and contrast levels were adjusted only after any quantification was performed and for presentational purposes only.

**Quantitative image measurements** When measurements were made between control and experimental samples, kidneys were stained in the same tube and in the same solution. Images were captured using the same settings and care was taken to ensure image intensity ranges were appropriate for comparisons to be made.

*Kidney growth in response to LY294002:* The area of each explants kidney was measured at the widest point of each kidney. The free-hand drawing tool in Fiji was used to trace the periphery of the kidneys. The portion of the ureteric bud stalk that projected outside the

kidney was not included in the measurements. Student's t-tests were used to compare conditions and p-values are displayed on graph in Figure 1B. 9 and 8 kidneys used for control and Ly294002 conditions, respectively.

*Measurements of nephron size in response to LY294002:* The width of nephron tubules were measured at the widest point of each tubule within the z-plane captured. Student's t-tests were used to compare conditions and p-values are displayed on graph in Figure 1C. 9 and 8 kidneys used for control and Ly294002 conditions, respectively.

*Measurements of nephron number, tip number, and Jag1$^{+}$area:* Kidneys stained for Wt1, Jag1, and Cdh1, were scanned on a confocal. Nephrons and tips were counted by eye on maximum intensity z-projections. JAG1 areas were measured by thresholding the images and using the Particle Analyser to measure individual nephrons. Student's t-tests were used to compare conditions. P-values are shown on Graphs in Figure SJ. A total of 12 kidneys were used, 6 for each condition.

**List of TaqMan Primers and Probes** (Target/Ensemble ID/Primer 1/Primer 2/UPL probe)
*Lhx1*/ENSMUSG00000018698/aatgcaacctgaccgagaag/cgcatttggtaccgaaacat/probe7,
*Bmp2*/ENSMUSG00000027358/cggactgcggtctcctaa/ggggaagcagcaacactaga/probe49,
*Jag1*/ENSMUSG00000027276/gaggcgtcctctgaaaaaca/acccaagccactgttaagaca/probe6,
*Wnt4*/ENSMUSG00000036856/ctggactccctccctgtctt/atgcccttgtcactgcaaa/probe62,
*Osr1*/ENSMUSG00000048387/agaagcgtcagaagtctagttcg/ggaaccgcaatgatttcaa/probe50,
*Six2*/ENSMUSG00000024134/caagtcagcaactggttcaaga/actgccattgagcgagga/probe5,
*Cited1*/ENSMUSG00000051159/gaggcctgcacttgatgtc/tggagtaggccagagagttca/probe12,
*Lef1*/ENSMUSG00000027985/tggttaacgagtccgaaatca/agaggacggggcttgtct/probe20,
*Axin2*/ENSMUSG00000000142/gagagtgagcggcagagc/cggctgactcgttctcct/probe96,
*Irx2*/ENSMUSG00000001504/gaagcaaggaggagagttcaga/gtgagcgagtcgacgtgtag/probe98,
*HeyL*/ENSMUSG00000032744/ctgaattgcgacgattggt/gcaagacctcagctttctcc/probe25,
*Lgr5*/ENSMUST00000020350.8/cttcactcggtgcagtgct/gatcagccagctaccaaatagg/probe60.

**Supplemental References**


Chen, B., Dodge, M.E., Tang, W., Lu, J., Ma, Z., Fan, C.W., Wei, S., Hao, W., Kilgore, J., Williams, N.S., *et al.* (2009). Small molecule-mediated disruption of Wnt-dependent signaling in tissue regeneration and cancer. Nature chemical biology *5*, 100-107.

Cuny, G.D., Yu, P.B., Laha, J.K., Xing, X., Liu, J.F., Lai, C.S., Deng, D.Y., Sachidanandan, C., Bloch, K.D., and Peterson, R.T. (2008). Structure-activity relationship study of bone morphogenetic protein (BMP) signaling inhibitors. Bioorganic & medicinal chemistry letters *18*, 4388-4392.

Davies, J. (1994). Control of Calbindin-D-28k Expression in Developing Mouse Kidney. Dev Dynam *199*, 45-51.

Davies, J.A., and Garrod, D.R. (1995). Induction of early stages of kidney tubule differentiation by lithium ions. Dev Biol *167*, 50-60.

Folkes, A.J., Ahmadi, K., Alderton, W.K., Alix, S., Baker, S.J., Box, G., Chuckowree, I.S., Clarke, P.A., Depledge, P., Eccles, S.A., *et al.* (2008). The identification of 2-(1H-indazol-4-yl)-6-(4-methanesulfonyl-piperazin-1-ylmethyl)-4-morpholin-4-yl-t hieno[3,2-d]pyrimidine (GDC-0941) as a potent, selective, orally bioavailable inhibitor of class I PI3 kinase for the treatment of cancer. J Med Chem *51*, 5522-5532.

Karner, C.M., Merkel, C.E., Dodge, M., Ma, Z., Lu, J., Chen, C., Lum, L., and Carroll, T.J. (2010). Tankyrase is necessary for canonical Wnt signaling during kidney development. Dev Dyn *239*, 2014-2023.

Meek, S., Wei, J., Sutherland, L., Nilges, B., Buehr, M., Tomlinson, S.R., Thomson, A.J., and Burdon, T. (2013). Tuning of beta-catenin activity is required to stabilize self-renewal of rat embryonic stem cells. Stem cells *31*, 2104-2115.

Ring, D.B., Johnson, K.W., Henriksen, E.J., Nuss, J.M., Goff, D., Kinnick, T.R., Ma, S.T., Reeder, J.W., Samuels, I., Slabiak, T., *et al.* (2003). Selective glycogen synthase kinase 3 inhibitors potentiate insulin activation of glucose transport and utilization in vitro and in vivo. Diabetes *52*, 588-595.

Vlahos, C.J., Matter, W.F., Hui, K.Y., and Brown, R.F. (1994). A specific inhibitor of phosphatidylinositol 3-kinase, 2-(4-morpholinyl)-8-phenyl-4H-1-benzopyran-4-one (LY294002). J Biol Chem *269*, 5241-5248.

Vogt, J., Traynor, R., and Sapkota, G.P. (2011). The specificities of small molecule inhibitors of the TGFss and BMP pathways. Cell Signal *23*, 1831-1842.

Workman, P., Clarke, P.A., Raynaud, F.I., and van Montfort, R.L. (2010). Drugging the PI3 kinome: from chemical tools to drugs in the clinic. Cancer Res *70*, 2146-2157.
