# Supplementary material for: The PI3K Pathway Balances Self-Renewal and Differentiation of Nephron Progenitor Cells through β-Catenin Signaling
Source: Stem Cell Reports. 2015 Mar 5;4(4):551–60. doi: 10.1016/j.stemcr.2015.01.021 (PMC4400645; doi:10.1016/j.stemcr.2015.01.021)
Supplement: Document S2. Article plus Supplemental Information [file mmc5.pdf]

Stem Cell Reports
Report
OPEN ACCESS

The PI3K Pathway Balances Self-Renewal and Differentiation of Nephron Progenitor Cells through β-Catenin Signaling

Nils Olof Lindström,[1,2,*] Neil Oliver Carragher,[2] and Peter Hohenstein[1,3,*]
[1]The Roslin Institute, University of Edinburgh, Easter Bush Campus, Midlothian EH25 9RG, UK
[2]Edinburgh Cancer Research Centre, MRC Institute of Genetics and Molecular Medicine, University of Edinburgh, Western General Hospital, Edinburgh EH4 2XR, UK
[3]MRC Human Genetics Unit, MRC Institute of Genetics and Molecular Medicine, University of Edinburgh, Western General Hospital, Edinburgh EH4 2XU, UK
*Correspondence: nils.lindstrom@roslin.ed.ac.uk (N.O.L.), peter.hohenstein@roslin.ed.ac.uk (P.H.)
http://dx.doi.org/10.1016/j.stemcr.2015.01.021
This is an open access article under the CC BY license (http://creativecommons.org/licenses/by/4.0/).

SUMMARY

Nephron progenitor cells differentiate to form nephrons during embryonic kidney development. In contrast, self-renewal maintains progenitor numbers and premature depletion leads to impaired kidney function. Here we analyze the PI3K pathway as a point of convergence for the multiple pathways that are known to control self-renewal in the kidney. We demonstrate that a reduction in PI3K signaling triggers premature differentiation of the progenitors and activates a differentiation program that precedes the mesenchymal-to-epithelial transition through ectopic activation of the β-catenin pathway. Therefore, the combined output of PI3K and other pathways fine-tunes the balance between self-renewal and differentiation in nephron progenitors.

INTRODUCTION

Embryonic nephron progenitor cells (ENPs) form a population of cells that gives rise to all nephrons in a kidney (Costantini and Kopan, 2010). The balance between maintenance of ENPs and differentiation of the cells to form nephrons is essential for the development of a functional kidney. Multiple genes and signaling pathways have been shown to be involved in controlling this balance, including β-catenin signaling (Park et al., 2007), *Six2* (Self et al., 2006), *Fgf9/20* (Barak et al., 2012), *Bmp7* (Brown et al., 2013), *Osr1* (Xu et al., 2014), and *Fodxd1/Hippo/Yap* (Das et al., 2013). It may be surprising that mutations in so many, and maybe more, pathways all lead to a disturbance of this control. Two models for the control of ENPs can be envisioned. In one, each of these signals or pathways has its own discrete function, each of which is essential to control the balance between self-renewal and differentiation. The other possibility would be close crosstalk between signals, and convergence into one or a limited number of pathways that controls this balance. Close cooperation among the β-catenin, SIX2, and OSR1 proteins in the direct regulation of transcription of ENP genes has been demonstrated (Karner et al., 2011; Park et al., 2012; Xu et al., 2014), but the incorporation of other signals into a concise control mechanism remains to be demonstrated.

A potential point of convergence of multiple signals is the PI3K pathway, which acts downstream of receptor tyrosine kinases (RTKs) and G protein-coupled receptors and is negatively controlled by PTEN (Carracedo and Pandolfi, 2008). FGFs act directly on RTKs, YAP controls the expression of *miR-29*, which in turn targets PTEN, thereby increasing PI3K signaling (Tumaneng et al., 2012), and BMPs can control the PI3K pathway to regulate β-catenin-mediated signaling (He et al., 2004). FGFs and BMPs can both activate the MAPK pathway, which can be further modulated by PI3K signaling (Lanner and Rossant, 2010). Moreover, PI3K signaling has been shown to be important in controlling self-renewal of hematopoietic and embryonic stem cells (Paling et al., 2004; Perry et al., 2011).

Not much is known about PI3K signaling in the developing kidney. It was shown to be critical downstream of the GDNF/Ret system for normal branching morphogenesis (Kim and Dressler, 2007; Tang et al., 2002). In the nephrogenic lineage, overexpression of *Spry1*, an antagonist of RTKs, results in reduced expression of *Six2* and *Cited1*, two ENP markers, while in vitro recombinant FGF briefly can keep isolated ENPs in a CITED1$^+$ state in a Ras/PI3K-dependent manner (Brown et al., 2011). We therefore set out to analyze the role of the PI3K pathway in the control of ENPs in more detail.

RESULTS

ENP Maintenance Requires PI3K Signaling

To examine the role of PI3K during ENP self-renewal and differentiation, we blocked PI3K with LY294002 in kidney organ cultures. Treatment of kidney rudiments with this compound for 48 hr visually disturbed branching of the ureteric bud as described before (Tang et al., 2002; Figure 1A). PI3K inhibition resulted in reduced kidney size, but nephrons with increased diameter (Figure 1B) and a thinning of the SIX2$^+$ ENP-containing cap mesenchyme

CrossMark

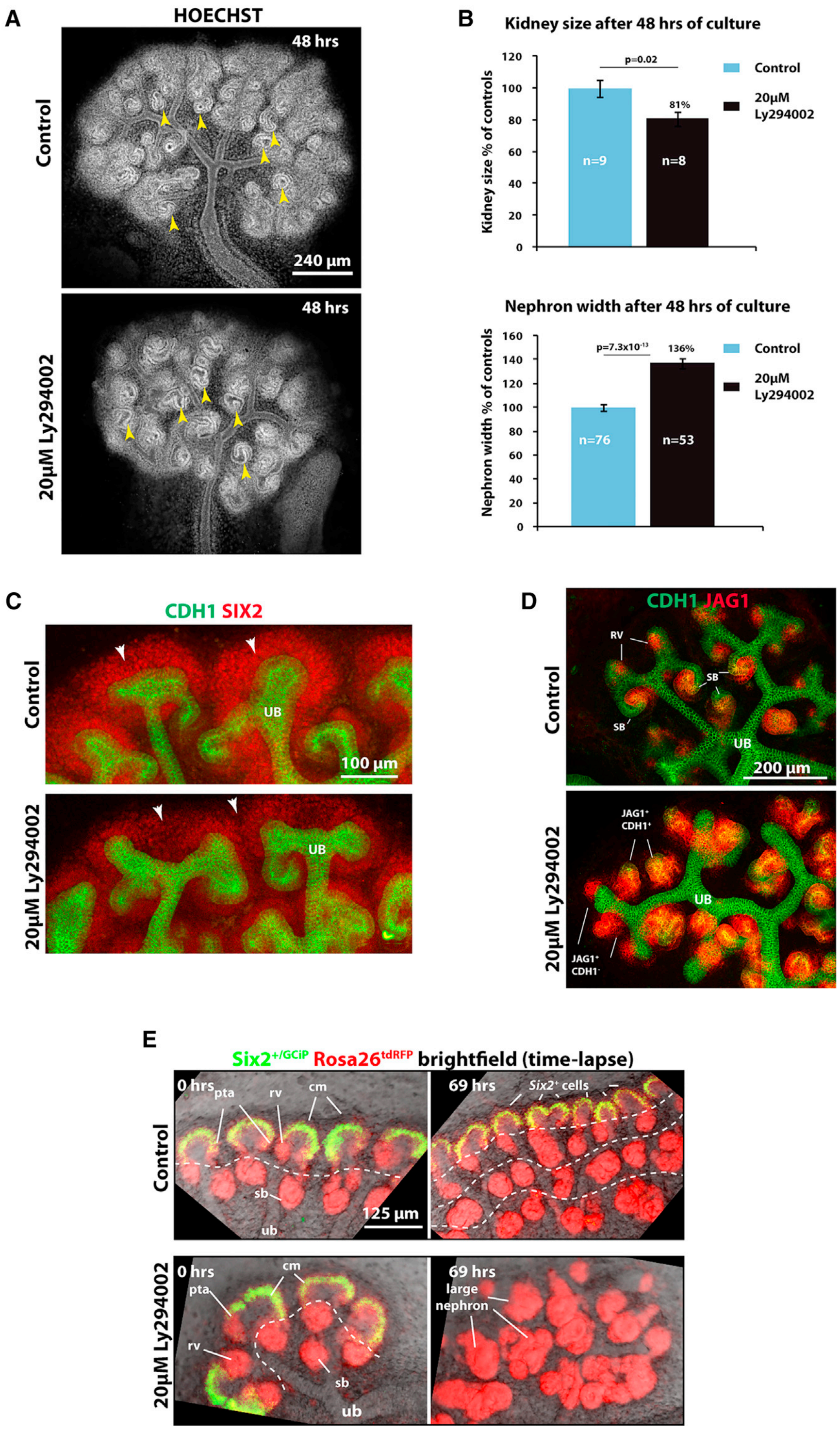

Figure 1. PI3K/Akt Signaling Is Necessary for ENP Self-Renewal and Kidney Development

(A) E12.5 kidneys cultured for 48 hr. Arrowheads point to nephrons.

(B) Measurements of kidney area and nephron tubule widths from kidneys. Nine and eight separate kidneys and 76 and 53 nephrons were analyzed for control and Ly294002 conditions, respectively. Error bars indicate SEM. Significance calculated using Student's t test.

(C and D) Kidneys cultured for 24 hr. Arrowheads indicate nephron progenitors.

(E) Time-lapse data for E11.5 *Six2*$^{+/GCiP}$; *Rosa26*^{tdRFP} kidneys cultured for 69 hr. White dashed line indicates generations of nephrons forming. Cm, cap mesenchyme containing ENPs; pta, pretubular aggregate; rv, renal vesicle; sb, s-shaped body; ub, ureteric bud; ubt, ureteric bud tip. Culture conditions and labeling are as indicated in figures. See also Figure S1.

(Figures 1A and 1C; Self et al., 2006) after blocking PI3K for just 24 hr, a time point when branching was still unaffected (not shown). We confirmed this phenotype using a second, structurally dissimilar PI3K inhibitor, GDC-0941 (Figures S1A–S1E; Table S1). Reduced staining for mTORC1 pSer2448 and AKT pSer473 confirmed the inhibition of the PI3K pathway by LY294002 (Figures S1F and S1G).

Ly294002 treatment of kidney rudiments for 24 hr resulted in the ectopic induction and formation of amorphous nephron structures (Figure 1D), suggesting an

important role for PI3K signaling in ENP maintenance. We crossed *Six2*$^{+/GCiP}$ mice expressing a CreGFP fusion from the endogenous *Six2* locus (Dolt et al., 2013) with *Rosa26*$^{+/tdRFP}$ Cre reporter mice (Luche et al., 2007) to label ENPs GFP$^+$/RFP$^+$, whereas their post-mesenchymal-to-epithelial transition (MET) descendants would be GFP$^-$/RFP$^+$ as *Six2* would no longer be expressed. Time-lapse analysis of cultured embryonic kidneys showed that, whereas under control conditions *Six2* expression is maintained throughout the course of the experiment, PI3K inhibition leads to a rapid exhaustion of the ENPs as they differentiated into GFP$^-$/RFP$^+$ structures (Figure 1E; Figures S1H and S1I; Movie S1). Nephrons that had formed before Ly294002 treatment grew exceedingly large. After 96 hr, Ly294002 treatment had reduced both ureteric bud branching and nephron formation (Figure S1J). The average size of JAG1$^+$ structures increased 6-fold. Although fewer nephrons formed, the total area of JAG1$^+$ structures per kidney increased 2.7-fold. While PI3K inhibition increased apoptosis in the kidney, the apoptotic cells were mainly found surrounding the ENPs (Figure S1K), not in SIX2$^+$ cells as found before (Motamedi et al., 2014). Combined, these data show that intact PI3K signaling is pivotal for the maintenance of ENPs.

Differentiation and Epithelialization Can Be Uncoupled

Current models for nephron development assume that mesenchymal ENPs undergo a MET before segment-specific expression programs are activated (Costantini and Kopan, 2010). We noted, however, that the ectopic nephrons that form under conditions of PI3K inhibition show signs of differentiation, for instance, expression of JAG1 and not all JAG1$^+$ cells being fully epithelialized, as shown by the lack of CDH1 expression (Figure 1D). We analyzed this further at the 24 hr time point before the ENP population differentiated fully using qRT-PCR on RNA from *Six2*$^{+/GCiP}$ *Rosa26*$^{+/tdRFP}$ kidneys (Figures S2A–S2C). This confirmed that, in ENPs (GFP$^+$/RFP$^+$), cell expression of the ENP markers *Six2* and *Cited1* did not change after PI3K inhibition, though expression of *Osr1*, a marker of intermediate mesoderm that is maintained in the ENP stage, was reduced (Figure 2A). In contrast, in the same cells, Ly294002 treatment resulted in an upregulation of induction markers *Wnt4*, *Lhx1*, and *Cdh1* (Figure 2B) and segment markers *Jag1*, *Dll1*, and *HeyL* (Figure 2C). Note that, although we detected a modest upregulation of *Cdh1* mRNA 24 hr after PI3K inhibition (Figure 2B), at the same time point there was no sign of CDH1 protein expression in SIX2$^+$ cells (Figure 1C). After 24 hr of PI3K inhibition, ectopic nephrons showed expression of JAG1 protein, while the tight junction marker ZO-1 and adherence junction protein β-catenin were increased in expression, but no CDH1 protein (MET marker, Figures 2D and 2E). After 48 hr in Ly2094002 expression of LEF1, PAX2 (induction markers), JAG1, and ZO-1 as well as CDH1 (Figures 2F–2H) confirmed that full MET eventually takes place in these structures. Expression of SIX2 was almost completely gone from cells that expressed CDH1 protein (data not shown).

PI3K Signaling Modulates Endogenous β-Catenin Activity in ENPs

The differentiation of ENPs is positively controlled by β-catenin activity in the ENPs in response to a WNT9B signal from the ureteric bud (Karner et al., 2011; Park et al., 2012). We used time-lapse analysis of the *TCF*/*Lef*::H2B-GFP β-catenin activity reporter mouse (Ferrer-Vaquer et al., 2010) to test the involvement of β-catenin in the ectopic nephrons obtained through PI3K inhibition. In control cultures, low-level activity of the reporter could be seen in the ENPs in the cap mesenchyme, and the signal increased in epithelialized CDH1$^+$ nephrons (Figure 3A, top; Movie S2; Figures S3A and S3B). Ly294002 treatment of reporter kidneys resulted in activity of the β-catenin-signaling pathway in the ectopic nephrons, and these GFP$^+$ structures later became CDH1$^+$ (Figure 3A, bottom; Movie S2). To test if β-catenin activity is required for PI3K inhibition-induced nephron induction, we used IWR1, a Tankyrase inhibitor that results in inhibition of β-catenin signaling. We confirmed that IWR1 specifically reduced β-catenin targets and the β-catenin-signaling reporter in kidneys, and IWR1 effectively blocked Ly294002-induced ectopic nephrons (Figure 3B; Figures S3C–S3E).

To determine if PI3K inhibition is sufficient for ectopic nephron induction, we tried to induce nephron induction in isolated mesenchymes, where the WNT9B induction signal from the ureteric bud is absent, by inhibiting PI3K activity (Figure 3C). Neither control conditions nor treatment with Ly294002 showed any signs of nephron induction, as monitored by WT1, CDH1, and JAG1 immunostaining. This showed PI3K inhibition is not sufficient for nephron induction. To test if PI3K inhibition can have an additive effect on β-catenin signaling in the process, we used GSK3β inhibitor CHIR99021 to activate β-catenin signaling. It is known that high β-catenin activity can induce nephron formation, but inhibits the subsequent epithelialization (Davies and Garrod, 1995; Kuure et al., 2007; Park et al., 2007). We therefore titrated the dose of CHIR to find a concentration that allowed induction and differentiation CHIR^{medium} (1.5 μM), and one just insufficient to trigger induction and differentiation CHIR^{low} (0.75 μM). The combination of CHIR^{low} with Ly294002 resulted in robust nephron epithelialization and differentiation but CHIR^{low} treatment alone did not (Figure 3C), demonstrating an additive and potentially synergistic effect of inhibiting PI3K signaling on the β-catenin-mediated

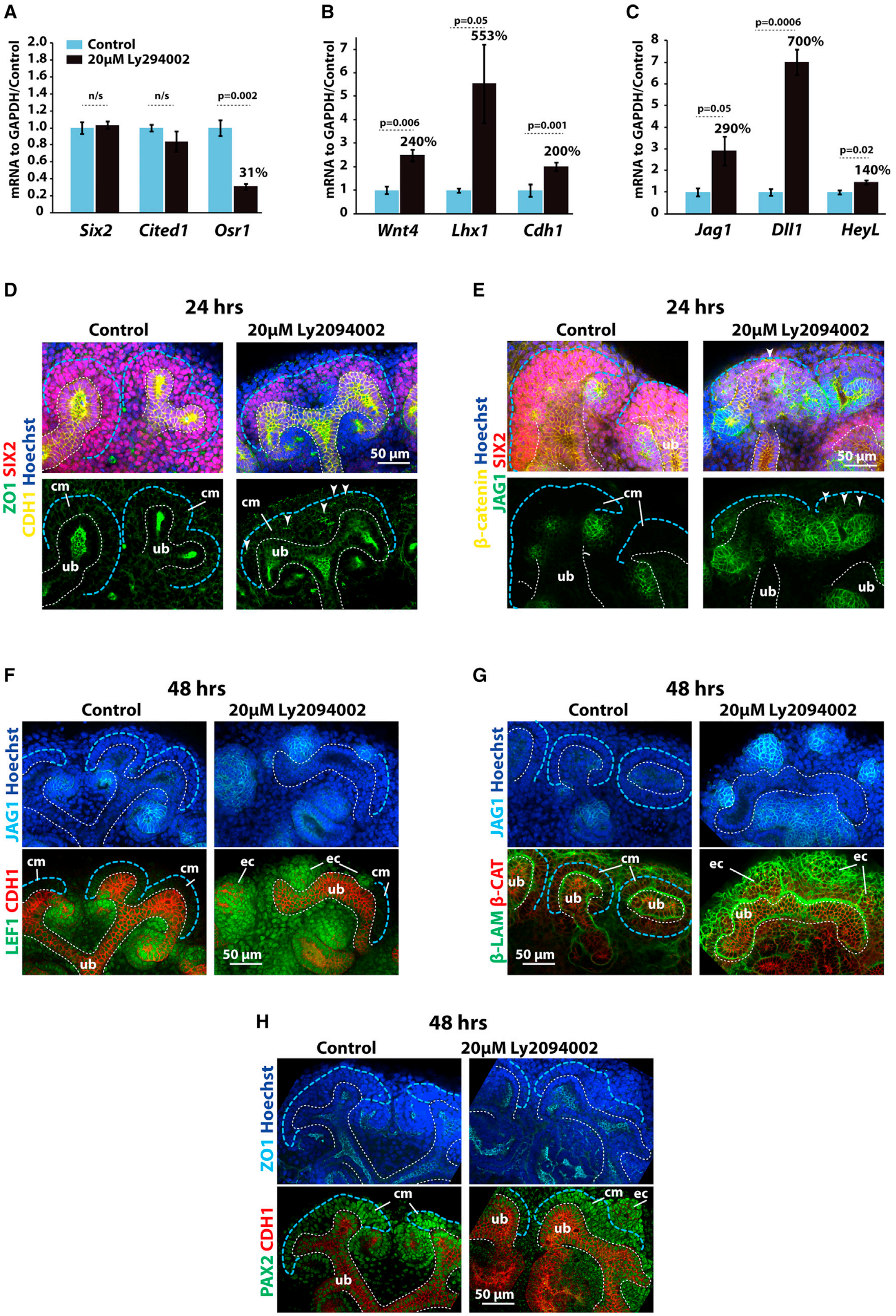

(legend on next page)

nephron induction process. Combining PI3K inhibition with CHIR^{medium} conditions led to massive epithelialization and differentiation far exceeding the effect of CHIR^{medium} alone, providing more evidence of this additive effect. We further confirmed this by staining for LEF1 and β-catenin protein, which were both upregulated by combined treatment with Ly294002 and CHIR, but not with Ly294002 on its own (Figure S3F). We also confirmed the synergistic effect with endogenous β-catenin signaling in intact kidneys with the ureteric bud still present (Figure 3D; Figure S4). After 24 hr of culture, we detected SIX2^{+}/JAG1^{+} cells, and ENPs strongly expressing β-catenin, ZO1, LEF1, and β-laminin. Although we identified β-catenin^{+} and ZO1^{+} foci, we did not detect CDH1 in these cells.

PI3K Signaling Interaction with Other Pathways

BMP7/pSMAD signaling can switch ENPs from self-renewing to differentiating (Brown et al., 2013). We compared the effects of inhibiting PI3K signaling to blocking BMP receptors with LDN-193189. Blocking BMP signaling led to a loss of SIX2^{+} cells, similar to that seen in *Bmp7*-deficient animals (Brown et al., 2013), and disruption of branching morphogenesis, but did not trigger ectopic nephron formation; inhibition of PI3K still drove ectopic nephron formation and altered the growth of nephrons when BMP signaling was inhibited (Figure 4A). Inhibition of BMP signaling did not trigger increased β-catenin signaling in ENPs (Figure 4B; Movie S3), but simultaneous inhibition of BMP signaling and activation of β-catenin actually resulted in massive and rapid upregulation of β-catenin signaling in ENPs. Inhibiting PI3K and activating β-catenin at the same time also resulted in massive upregulation of β-catenin signaling in ENPs, but the dynamics of the ENP response to CHIR + LDN-193189 and CHIR + Ly294002 were different. ENPs with only CHIR activated β-catenin signaling slower than those in CHIR + LDN-193189, and the cells were more motile and migrated away from the ureteric bud tips. In CHIR + LDN-193189, β-catenin signaling was activated very quickly, to higher levels, and the cells remained surrounding the ureteric bud tips. In CHIR + Ly294002 conditions, β-catenin signaling was activated quicker than in CHIR-only conditions, but, similar to CHIR + LDN-193189 conditions, however, cells displayed motility (Movie S3). Although CHIR + LDN-193189 and CHIR + Ly294002 created distinct responses from each other, they both increased the activation of β-catenin signaling compared to CHIR.

DISCUSSION

The data presented here support a role for PI3K signaling in controlling the balance between self-renewal and differentiation of ENPs. We showed that blocking the pathway with two structurally unrelated inhibitors leads to a rapid exhaustion of SIX2^{+} ENPs, as they differentiate and form large amorphous ectopic nephrons consisting of excessive numbers of differentiating ENPs. The cells differentiate directly where they are located and form nephron structures at positions around the whole of the ureteric bud tips (see lineage analyses of the ENPs in Movie S1). Our data show that PI3K function is coupled to the well-known role of β-catenin signaling in ENP balance control, but the differences we find between inhibiting PI3K and activating β-catenin show that PI3K signaling has additional β-catenin-independent roles as well. Our data on the combined inhibition of PI3K, BMPR, and GSK3β support a model where these pathways communicate with one another to regulate the stemness and differentiation of ENPs. The relative strengths of these pathways appear essential in this process, suggesting that self-renewal and differentiation are fine-tuned processes and not controlled by simple on/off control mechanisms.

A role for PI3K controlling ENPs fits well with current ideas of how FGF, BMP, β-catenin, and FAT4 regulate ENP self-renewal and differentiation (Figure 4C). The ENPs all receive the differentiation promoting β-catenin signal from the ureteric bud, but only the cells that receive a stromal signal differentiate (Das et al., 2013). The FAT4 signal from the stromal cells triggers phosphorylation and removal of YAP from the nucleus, thereby altering the transcriptional output of β-catenin (Das et al., 2013). As in other cell types, YAP signaling was shown to result in low levels of *miR-29*, increased PTEN, and inhibition of PI3K (Tumaneng et al., 2012). A model can be envisioned in which also this part of ENP control is fine-tuned by PI3K; however, this needs additional experimental verification.

Figure 2. Nephron Progenitors Differentiate into Nephrons when PI3K Is Inhibited

(A–C) qRT-PCR analyses on FACS-sorted cells from dissociated E12.5 $Six2^{+/GCiP}$;$Rosa26^{tdRFP}$ kidneys cultured for 24 hr. Cells from three kidneys were grouped to form each mRNA isolate replicate. Experiments were performed in triplicate with nine kidneys per treatment. All error bars indicate SEM. P values calculated using Student's t test.

(D and E) E12.5 kidneys cultured for 24 hr.

(F–H) E12.5 kidneys cultured for 48 hr. Blue dashed line surrounds the nephron progenitor cells; white dashed line surrounds the ureteric bud; white arrowheads indicate points of ectopic expression. Cm, cap mesenchyme; ub, ureteric bud; ec, ectopic nephron. Culture conditions and labeling are as indicated in figures. See also Figure S2.

©2015 The Authors

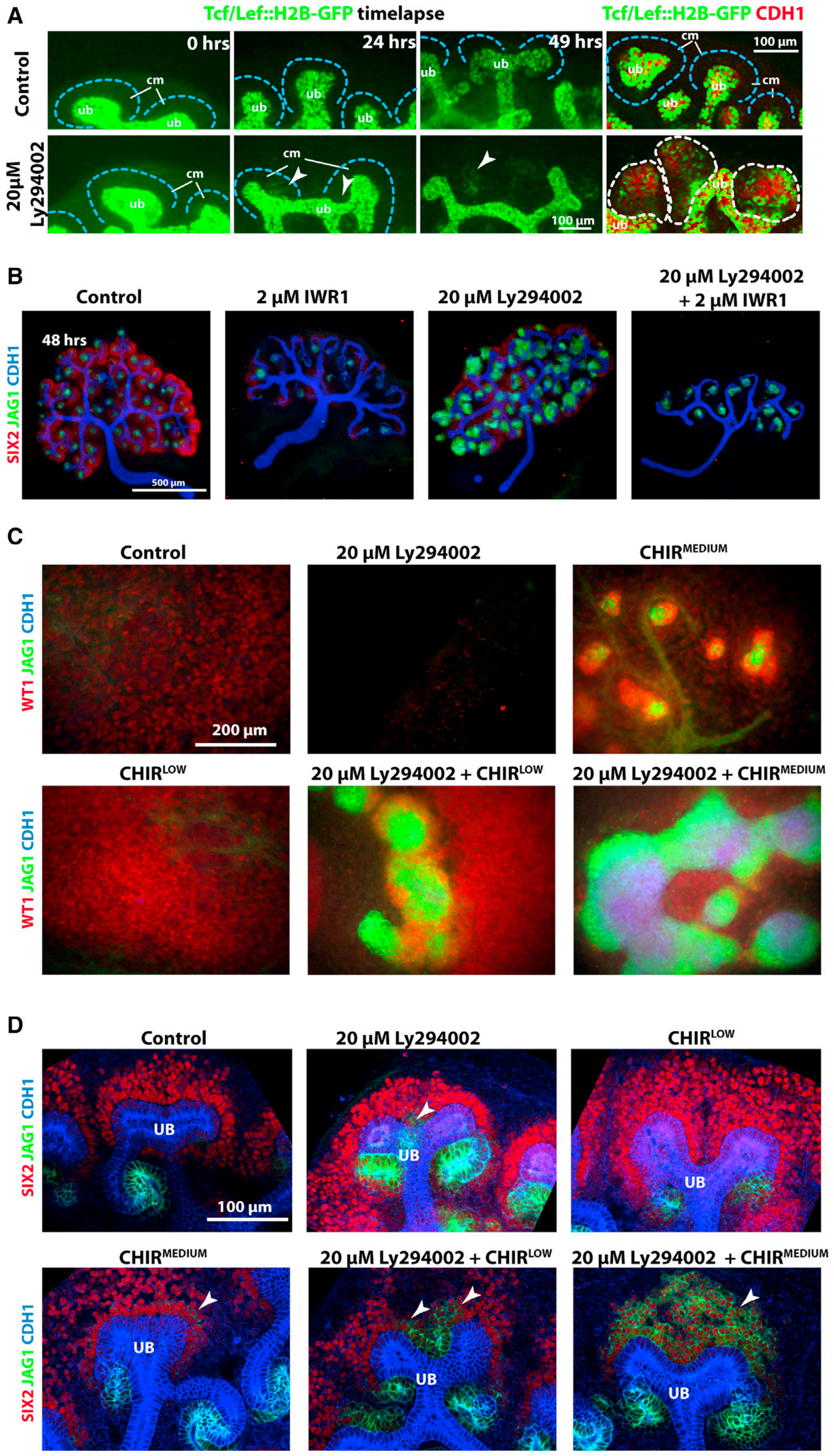

Figure 3. PI3K Inhibition Results in Ectopic Activation of β-cateninTcf/Lef Signaling in Nephron Progenitor Cells

(A) Time-lapse data showing E12.5 *TCF/Lef::H2B-GFP* kidneys cultured for 49 hr. (Right) Fixed and stained kidneys. Blue dashed line surrounds the nephron progenitor cells; white arrowheads indicate points of ectopic sites of GFP expression; white dashed line outlines ureteric bud epithelium and indicates ectopic GFP expression also positive for Cdh1.

(B) E12.5 kidneys cultured for 48 hr.

(C) Isolated mesenchyme cultured without the ureteric bud.

(D) Regions of ENPs and UBTs from whole kidneys cultured for 24 hr. Arrowheads indicate sites of ectopic expression. Cm, cap mesenchyme; ub, ureteric bud. Culture conditions and labeling are as indicated in figures. See also Figures S3 and S4.

In embryonic stem cells, PI3K inhibition has been shown to reduce β-catenin phosphorylation (Paling et al., 2004). In line with these findings, in our hands, inhibition of PI3K resulted in β-catenin signaling and simultaneous activation of β-catenin, and inhibition of PI3K had an additive effect on β-catenin activity. BMP signalling has previously been shown to be necessary for ENPs to respond to β-catenin activation in older kidneys, whereas cells from younger

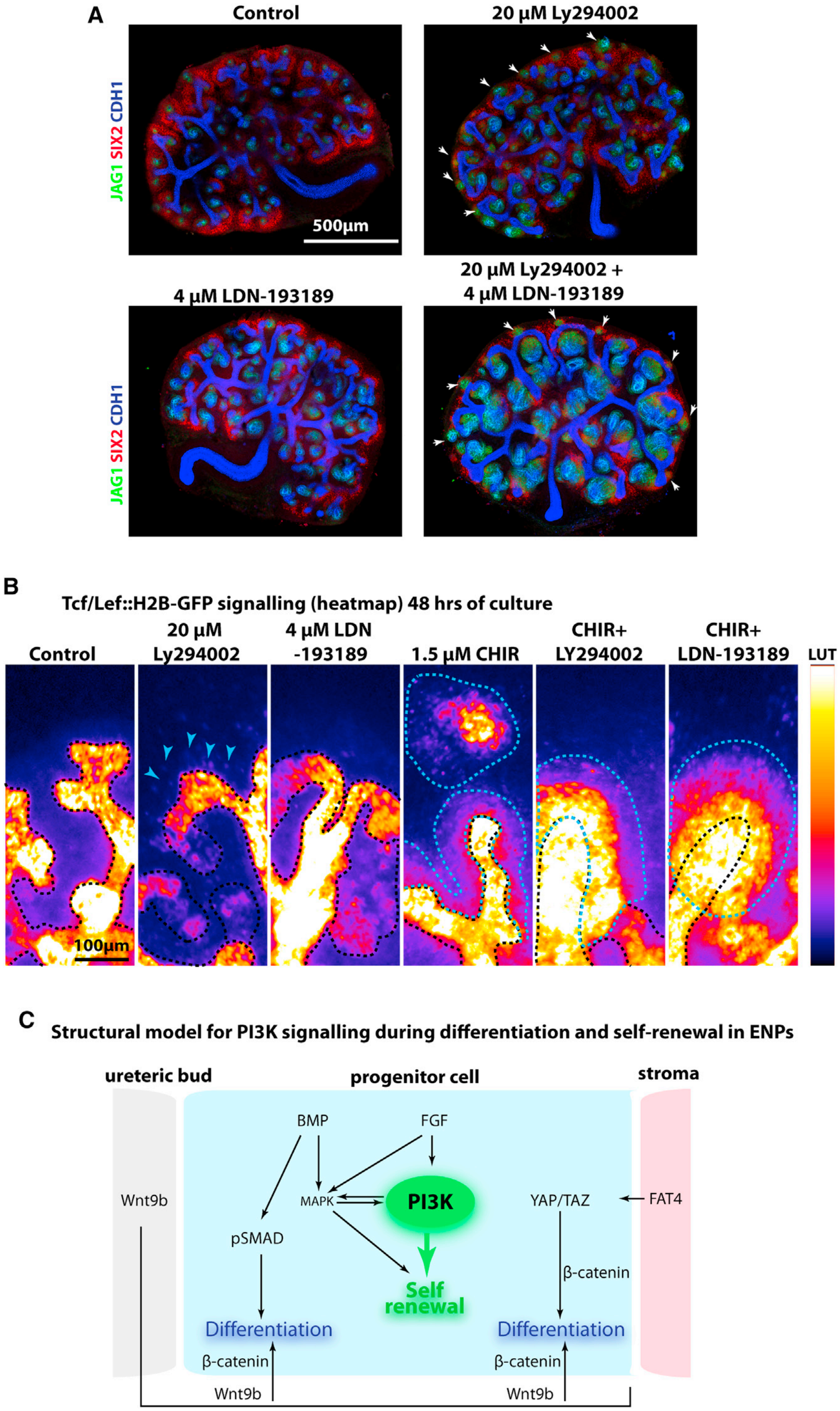

Figure 4. Multiple Signaling Pathways Feed into PI3K-Dependent ENP Self-Renewal

(A) E12.5 kidneys cultured for 48 hr. White arrowheads indicate ectopic ENP differentiation.

(B) Time-lapse data showing E12.5 *TCF/Lef::H2B-GFP* kidneys cultured for 48 hr. The GFP signal is shown as a heat map. Blue arrowheads indicate ectopic GFP^+ nuclei; black dashed line outlines the ureteric bud and normally positioned nephrogenic epithelium; blue dashed line outlines ectopic regions with strong GFP signal.

(C) Schematic model for PI3K signaling in ENP cells. The relationship of different signaling pathways is depicted and related to their outcomes in ENP cells. Culture conditions and labeling are as indicated in figures.

kidneys do not need this (Brown et al., 2013). In E12.5 kidneys, as used here, inhibition of BMP signaling did not on its own lead to ectopic nephron formation nor activation of β-catenin signaling. However, it was still possible to drive β-catenin signaling by inhibiting GSK3β, confirming that BMP signaling is not necessary for the ENPs to be able to signal via β-catenin during early kidney development. The dynamics of the ENP response to this dual inhibition/activation of BMPR and GSK3β was clearly different from that seen when β-catenin was activated on its own with the GSK3β inhibitor. This suggests that BMP signaling must still be controlling the induction process at this time

point in kidney development, but perhaps via a different mechanism. Likewise, the dynamics of inhibiting PI3K signaling together with GSK3β was different from just altering GSK3β on its own or simultaneously blocking BMP and GSK3β. This again confirms that PI3K is not solely acting as a regulator of β-catenin signaling.

Although the different pathways that we investigated possibly converge to regulate PI3K signaling, they clearly also control separate processes. BMPR inhibition on its own did not trigger ectopic ENP differentiation, but when either BMPR or PI3K inhibitors were applied together with the GSK3β inhibitor, they both produced additive but still distinguishable effects. It recently has been shown that BMP and FGF signaling could be interacting in an antagonistic balance, where FGF promotes ENP survival and BMP/SMAD signaling controls apoptosis, and WT1 regulates both protein pathways (Motamedi et al., 2014). Untangling the very complex interactions among these pathways will require a significant effort in the future, and our study exemplifies the need to gently modify, rather than obliterate, signaling pathways, as can be achieved through careful use of inhibitors instead of full gene knockouts (Davies, 2009).

Unexpectedly, we found expression of nephron segmentation and epithelialization markers prior to the formation of CDH^+ adherence junctions and the formation of a structurally distinct epithelium in PI3K-inhibited kidneys. We found that tight junctions began to form ($ZO\text{-}1^+$) and $\beta\text{-catenin}^+$ foci started to assemble, indicative of the formation of adherens junctions. Several other cadherins are expressed by the ENPs and could explain how $\beta\text{-catenin}^+$ foci assembled without CDH1 (Goto et al., 1998; Klein et al., 1988). Further evidence of the ENPs beginning to epithelialize comes from the cells depositing a basement membrane ($\beta\text{-laminin}^+$). Although, admittedly, inhibition of PI3K and simultaneous activation of β-catenin signaling does not necessarily reflect the normal situation, it does emphasize that shifts between the mesenchymal and epithelial states are fluid and dynamic transitions rather than sudden shifts. Indeed, while CDH1 expression can be detected only after the initial aggregation during nephrogenesis (Vestweber et al., 1985), proximal nephron marker and adhesion protein CDH6 can be detected in mesenchymal cells before CDH1 is detected (Cho et al., 1998). We have suggested previously that nephron segmentation starting before the formation of a rigid epithelium could explain the patterning defects in nephrons with reduced Rho-kinase activity (Lindström et al., 2013). ENPs can express genes associated with differentiation and segmentation prior to nephron formation (Brunskill et al., 2014). A better description of the dynamics of the renal MET is clearly necessary, and not just for semantic reasons. Understanding when markers are first expressed will help the phenotypic description of kidney development. Moreover, every step in the MET process is a potential moment when phenotypes can arise under experimental conditions, as shown here, or in disease situations.

EXPERIMENTAL PROCEDURES

Extended details outlining specific steps and protocols can be found in the Supplemental Experimental Procedures.

Ethics Statement for Experimental Animals

All animal experiments were approved by the Edinburgh University Animal Welfare and Ethical Review Body, performed at the University of Edinburgh (UK), and carried out according to regulations specified by the Home Office and Project Licenses 60/3788 and 60/4473.

Experimental Animals

For timed matings, noon of the day a vaginal plug was found was considered E0.5. CD1 animals were purchased from Charles River Laboratories. *TCF/Lef:H2B-EGFP* (Tg(TCF/Lef1-HIST1H2BB/EGFP)61Hadj) (Ferrer-Vaquer et al., 2010) were crossed with CD1s. *Six2*$^{+/GCiP}$ (Dolt et al., 2013) mice were crossed with Rosa26^{tdRFP} (Gt(ROSA)26Sor^{tm1Hjf}) (Luche et al., 2007).

Organ Culture and Time Lapse

E12.5 kidneys were used. Kidney cultures were performed as described previously (Lindström et al., 2013). Isolated E11.5 mesenchyme was collected and induced as described previously (Davies and Garrod, 1995).

Fluorescence-Activated Cell Sorting Analyses for Cell Analyses and RNA Isolation

Six2$^{+/GCiP}$;*Rosa26t*^{dRFP} kidneys were dissociated into single cells and sorted for GFP and RFP using a FACSAriaIIIu (Becton Dickinson).

RNA Analysis

RNA was isolated using RNeasy micro kits (QIAGEN). cDNA was generated using Superscript III and random primers. For the TaqMan reactions LightCycler 480 Probes Master (Roche) kits were used. Gene-specific primers and probes were designed using Ensemble IDs and the Roche Universal ProbeLibrary Assay Design Center. PCRs were multiplexed with *Gapdh* as an internal reference. Primer and probes are listed in the Supplemental Experimental Procedures. Pharmaceutical inhibitors are listed in Table S1. Inhibitors were used as specified in the text.

Immunofluorescent Staining

Kidneys were fixed in −20°C methanol or in 4% paraformaldehyde (PFA) in 1×PBS for 20 min followed by −20°C methanol when fluorescent proteins were present. Primary and secondary antibody incubations were performed with antibodies diluted into 1×PBS at 4°C O/N. See list of antibodies and extended protocol in the Supplemental Experimental Procedures.

Microscopy

Microscopy was performed on a Nikon TiE with 4×–10× objectives, or a Nikon A1R, N-STORM/A1, or a Zeiss LSM710 with 10× –63× objectives. Additional details can be found in the Supplemental Experimental Procedures. Quantitative image measurements are described in detail in the Supplemental Experimental Procedures.

SUPPLEMENTAL INFORMATION

Supplemental Information includes Supplemental Experimental Procedures, four figures, one table, and three movies and can be found with this article online at http://dx.doi.org/10.1016/j.stemcr.2015.01.021.

AUTHOR CONTRIBUTIONS

N.O.L performed and analyzed all experiments. N.O.L. and P.H. designed the experiments. N.O.L prepared figures. N.C. advised on small molecule usage. N.O.L and P.H. wrote the manuscript.

ACKNOWLEDGMENTS

We thank the members of the Hohenstein lab for valuable discussions. We thank Shahida Sheraz and Anna Thornburn for help with animals, and Bob Fleming, Matt Pearson, Paul Perry, and Ann Wheeler for advice on microscopy and fluorescence-activated cell sorting (FACS). N.O.L was supported by the National Centre for Replacement, Refinement and Reduction of Animals in Research (Grant 94808). The Roslin Institute receives Institute Strategic Programme Grant funding from the Biotechnology and Biological Sciences Research Council (BB/J004316/1).

Received: August 14, 2014
Revised: January 28, 2015
Accepted: January 29, 2015
Published: March 5, 2015

REFERENCES

Barak, H., Huh, S.H., Chen, S., Jeanpierre, C., Martinovic, J., Parisot, M., Bole-Feysot, C., Nitschké, P., Salomon, R., Antignac, C., et al. (2012). FGF9 and FGF20 maintain the stemness of nephron progenitors in mice and man. Dev. Cell *22*, 1191–1207.

Brown, A.C., Adams, D., de Caestecker, M., Yang, X., Friesel, R., and Oxburgh, L. (2011). FGF/EGF signaling regulates the renewal of early nephron progenitors during embryonic development. Development *138*, 5099–5112.

Brown, A.C., Muthukrishnan, S.D., Guay, J.A., Adams, D.C., Schafer, D.A., Fetting, J.L., and Oxburgh, L. (2013). Role for compartmentalization in nephron progenitor differentiation. Proc. Natl. Acad. Sci. USA *110*, 4640–4645.

Brunskill, E.W., Park, J.S., Chung, E., Chen, F., Magella, B., and Potter, S.S. (2014). Single cell dissection of early kidney development: multilineage priming. Development *141*, 3093–3101.

Carracedo, A., and Pandolfi, P.P. (2008). The PTEN-PI3K pathway: of feedbacks and cross-talks. Oncogene *27*, 5527–5541.

Cho, E.A., Patterson, L.T., Brookhiser, W.T., Mah, S., Kintner, C., and Dressler, G.R. (1998). Differential expression and function of cadherin-6 during renal epithelium development. Development *125*, 803–812.

Costantini, F., and Kopan, R. (2010). Patterning a complex organ: branching morphogenesis and nephron segmentation in kidney development. Dev. Cell *18*, 698–712.

Das, A., Tanigawa, S., Karner, C.M., Xin, M., Lum, L., Chen, C., Olson, E.N., Perantoni, A.O., and Carroll, T.J. (2013). Stromal-epithelial crosstalk regulates kidney progenitor cell differentiation. Nat. Cell Biol. *15*, 1035–1044.

Davies, J. (2009). Regulation, necessity, and the misinterpretation of knockouts. BioEssays *31*, 826–830.

Davies, J.A., and Garrod, D.R. (1995). Induction of early stages of kidney tubule differentiation by lithium ions. Dev. Biol. *167*, 50–60.

Dolt, K.S., Lawrence, M.L., Miller-Hodges, E., Slight, J., Thornburn, A., Devenney, P.S., and Hohenstein, P. (2013). A universal vector for high-efficiency multi-fragment recombineering of BACs and knock-in constructs. PLoS ONE *8*, e62054.

Ferrer-Vaquer, A., Piliszek, A., Tian, G., Aho, R.J., Dufort, D., and Hadjantonakis, A.K. (2010). A sensitive and bright single-cell resolution live imaging reporter of Wnt/ß-catenin signaling in the mouse. BMC Dev. Biol. *10*, 121.

Goto, S., Yaoita, E., Matsunami, H., Kondo, D., Yamamoto, T., Kawasaki, K., Arakawa, M., and Kihara, I. (1998). Involvement of R-cadherin in the early stage of glomerulogenesis. J. Am. Soc. Nephrol. *9*, 1234–1241.

He, X.C., Zhang, J., Tong, W.G., Tawfik, O., Ross, J., Scoville, D.H., Tian, Q., Zeng, X., He, X., Wiedemann, L.M., et al. (2004). BMP signaling inhibits intestinal stem cell self-renewal through suppression of Wnt-beta-catenin signaling. Nat. Genet. *36*, 1117–1121.

Karner, C.M., Das, A., Ma, Z., Self, M., Chen, C., Lum, L., Oliver, G., and Carroll, T.J. (2011). Canonical Wnt9b signaling balances progenitor cell expansion and differentiation during kidney development. Development *138*, 1247–1257.

Kim, D., and Dressler, G.R. (2007). PTEN modulates GDNF/RET mediated chemotaxis and branching morphogenesis in the developing kidney. Dev. Biol. *307*, 290–299.

Klein, G., Langegger, M., Goridis, C., and Ekblom, P. (1988). Neural cell adhesion molecules during embryonic induction and development of the kidney. Development *102*, 749–761.

Kuure, S., Popsueva, A., Jakobson, M., Sainio, K., and Sariola, H. (2007). Glycogen synthase kinase-3 inactivation and stabilization of beta-catenin induce nephron differentiation in isolated mouse and rat kidney mesenchymes. J. Am. Soc. Nephrol. *18*, 1130–1139.

Lanner, F., and Rossant, J. (2010). The role of FGF/Erk signaling in pluripotent cells. Development *137*, 3351–3360.

Lindström, N.O., Hohenstein, P., and Davies, J.A. (2013). Nephrons require Rho-kinase for proximal-distal polarity development. Sci. Rep. *3*, 2692.

Luche, H., Weber, O., Nageswara Rao, T., Blum, C., and Fehling, H.J. (2007). Faithful activation of an extra-bright red fluorescent

protein in "knock-in" Cre-reporter mice ideally suited for lineage tracing studies. Eur. J. Immunol. *37*, 43–53.

Motamedi, F.J., Badro, D.A., Clarkson, M., Lecca, M.R., Bradford, S.T., Buske, F.A., Saar, K., Hübner, N., Brändli, A.W., and Schedl, A. (2014). WT1 controls antagonistic FGF and BMP-pSMAD pathways in early renal progenitors. Nat. Commun. *5*, 4444.

Paling, N.R.D., Wheadon, H., Bone, H.K., and Welham, M.J. (2004). Regulation of embryonic stem cell self-renewal by phosphoinositide 3-kinase-dependent signaling. J. Biol. Chem. *279*, 48063–48070.

Park, J.S., Valerius, M.T., and McMahon, A.P. (2007). Wnt/beta-catenin signaling regulates nephron induction during mouse kidney development. Development *134*, 2533–2539.

Park, J.S., Ma, W., O'Brien, L.L., Chung, E., Guo, J.J., Cheng, J.G., Valerius, M.T., McMahon, J.A., Wong, W.H., and McMahon, A.P. (2012). Six2 and Wnt regulate self-renewal and commitment of nephron progenitors through shared gene regulatory networks. Dev. Cell *23*, 637–651.

Perry, J.M., He, X.C., Sugimura, R., Grindley, J.C., Haug, J.S., Ding, S., and Li, L. (2011). Cooperation between both Wnt/beta-catenin and PTEN/PI3K/Akt signaling promotes primitive hematopoietic stem cell self-renewal and expansion. Genes Dev. *25*, 1928–1942.

Self, M., Lagutin, O.V., Bowling, B., Hendrix, J., Cai, Y., Dressler, G.R., and Oliver, G. (2006). Six2 is required for suppression of nephrogenesis and progenitor renewal in the developing kidney. EMBO J. *25*, 5214–5228.

Tang, M.J., Cai, Y., Tsai, S.J., Wang, Y.K., and Dressler, G.R. (2002). Ureteric bud outgrowth in response to RET activation is mediated by phosphatidylinositol 3-kinase. Dev. Biol. *243*, 128–136.

Tumaneng, K., Schlegelmilch, K., Russell, R.C., Yimlamai, D., Basnet, H., Mahadevan, N., Fitamant, J., Bardeesy, N., Camargo, F.D., and Guan, K.L. (2012). YAP mediates crosstalk between the Hippo and PI(3)K–TOR pathways by suppressing PTEN via miR-29. Nat. Cell Biol. *14*, 1322–1329.

Vestweber, D., Kemler, R., and Ekblom, P. (1985). Cell-adhesion molecule uvomorulin during kidney development. Dev. Biol. *112*, 213–221.

Xu, J., Liu, H., Park, J.S., Lan, Y., and Jiang, R. (2014). Osr1 acts downstream of and interacts synergistically with Six2 to maintain nephron progenitor cells during kidney organogenesis. Development *141*, 1442–1452.

Stem Cell Reports, Volume *4*

Supplemental Information

The PI3K Pathway Balances Self-Renewal and Differentiation of Nephron Progenitor Cells through β-Catenin Signaling

Nils Olof Lindström, Neil Oliver Carragher, and Peter Hohenstein

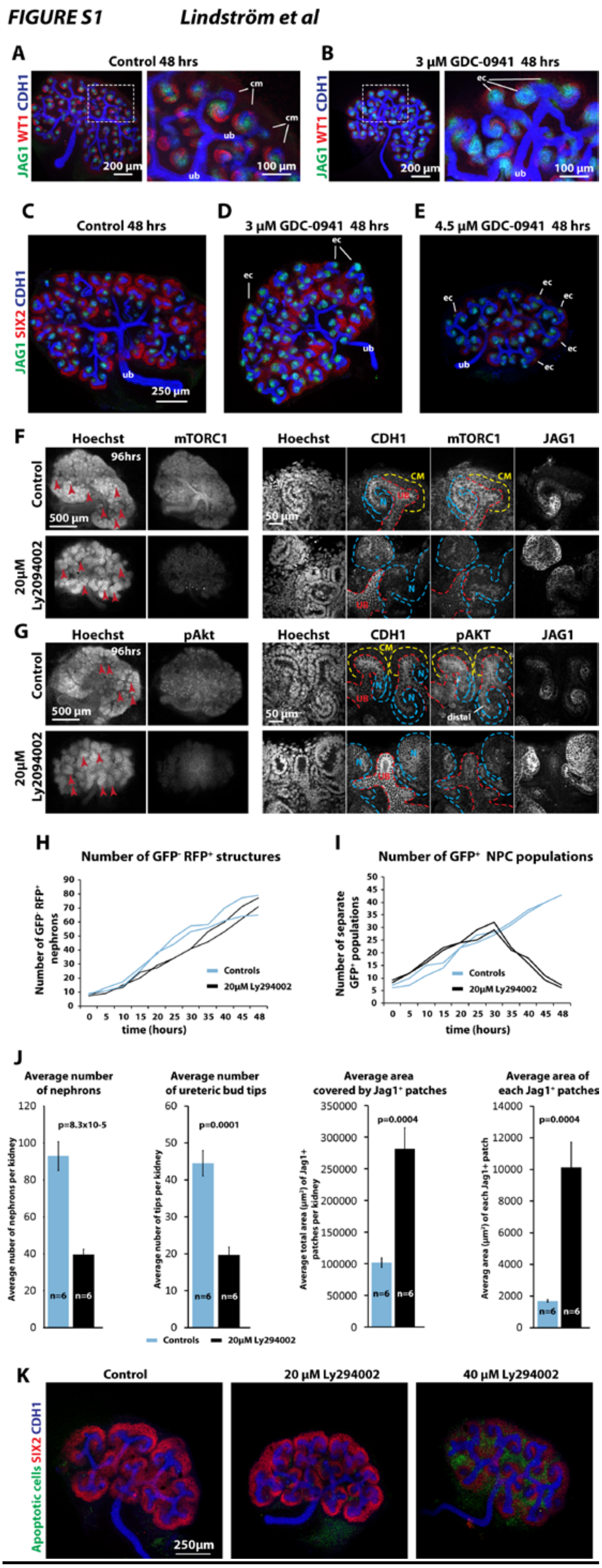
FIGURE S1 Lindström et al
A
Control 48 hrs
JAG1 WT1 CDH1
200 µm
100 µm
B
3 µM GDC-0941 48 hrs
C
Control 48 hrs
D
3 µM GDC-0941 48 hrs
E
4.5 µM GDC-0941 48 hrs
JAG1 SIX2 CDH1
250 µm
F
Hoechst
mTORC1
CDH1
JAG1
Control
20µM Ly2094002
96hrs
500 µm
50 µm
G
Hoechst
pAkt
pAKT
H
Number of GFP- RFP+ structures
Number of GFP- RFP+ nephrons
time (hours)
Controls
20µM Ly294002
I
Number of GFP+ NPC populations
Number of separate GFP+ populations
J
Average number of nephrons
p=8.3x10-5
Average nuber of nephrons per kidney
Average number of ureteric bud tips
p=0.0001
Average nuber of tips per kidney
Average area covered by Jag1+ patches
p=0.0004
Average total area (µm2) of Jag1+ patches per kidney
Average area of each Jag1+ patches
Averag area (µm2) of each Jag1+ patch
n=6
K
Control
20 µM Ly294002
40 µM Ly294002
Apoptotic cells SIX2 CDH1
250µm

FIGURE S2 *Lindström et al*

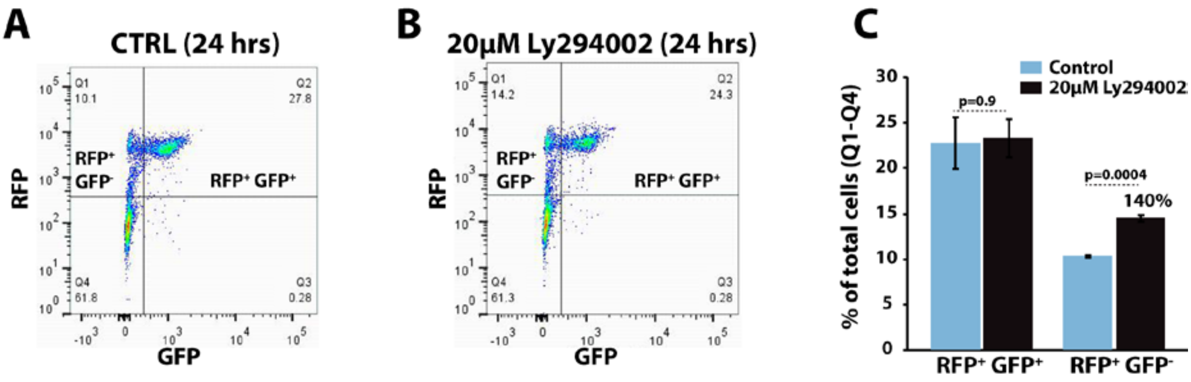

FIGURE S3 *Lindström et al*

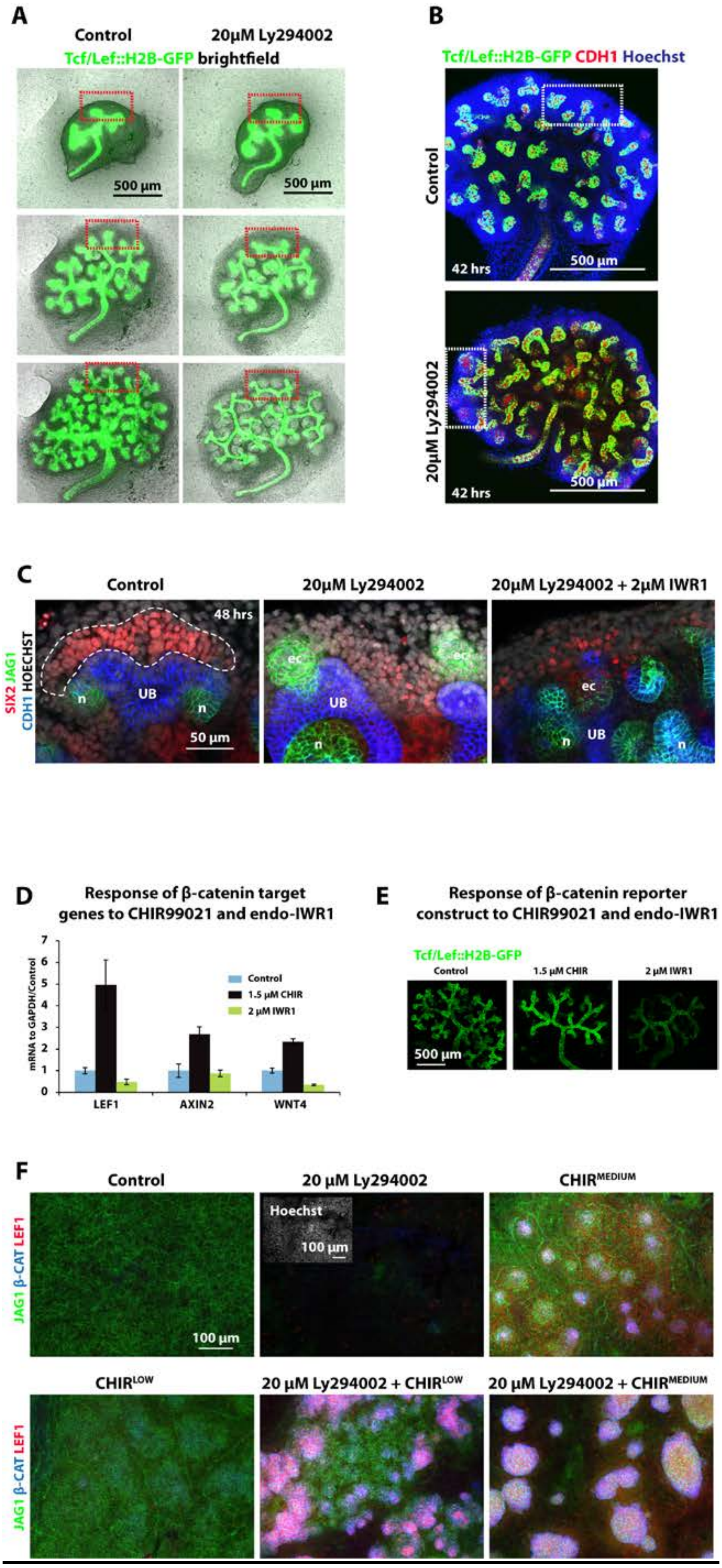

FIGURE S4   *Lindström et al*

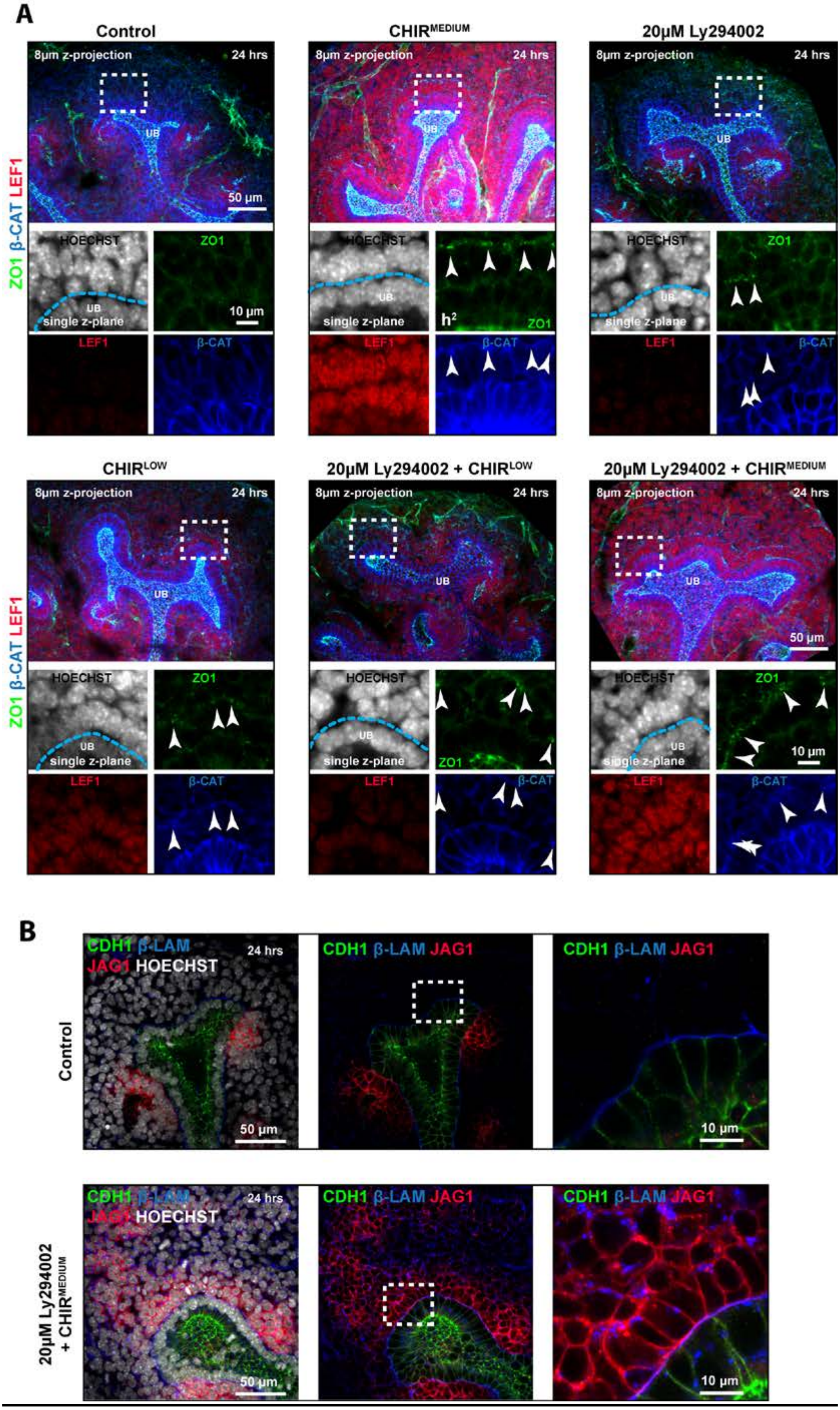

Supplementary Figure legends

Figure S1 – Relating to Figure 1. PI3K/Akt signalling is necessary for normal kidney development.

(**A-E**) Kidneys cultured for 48 hrs - square boxed indicate magnified areas shown. (**F-G**) E12.5 kidneys and magnified areas cultured for 96 hrs. Red arrowheads point to nephron tubules. Blue dashed lines indicate nephron structures. Red dashed lines indicate ureteric bud structures. Yellow dashed line indicates nephron progenitor cells. (**H-I**) Graphs showing time-lapse data from $Six2^{+/GCiP}$ *; Rosa26*^{tdRFP} kidneys. (**H**) Graph showing the number of nephrons that formed over time. (**I**) Graph showing the number of separate nephron progenitor populations at each time-point. (**J**) Graphs showing average number of nephrons and ureteric bud tips per kidney, and average area of JAG^{+} expressing patches and average area per kidney covered by $JAG1^{+}$ patches. Measurements made on kidneys after 96 hrs of culture. 6 kidneys were analysed per condition. Error bars indicate SEM. Significance calculated using Student's t-tests. (**K**) E12.5 kidneys cultured for 48 hrs. Ub- ureteric bud, ec- ectopic nephron. Culture conditions and labelling are as indicated in figures.

Figure S2 – Relating to Figure 2. Isolation of ENPs from $Six2^{+/GCiP}$; $Rosa26^{tdRFP}$ kidneys.

(**A-B**) FACS graphs of sorted E12.5 $Six2^{+/GCiP}$ *; Rosa26*^{tdRFP}. (**C**) Cell numbers as a percentage of the total. 9 kidneys were used per treatment. Error bars indicate SEM. Significance calculated using Student's t-tests.

Figure S3 – Relating to Figure 3. PI3K inhibition results in ectopic activation of β-cateninTcf/Lef signalling in nephron progenitor cells.

(**A-B**) Time-lapse data and immunofluorescent stains showing E12.5 *TCF/Lef::H2B-GFP* kidneys cultured for 49 hrs. White and red squares indicate magnified areas displayed in (Figure 3A). (**C**) Regions of kidneys shown – relating to Figure 3B. (**D**) qRT-PCR analyses on mRNA isolated from whole E12.5 kidneys cultured for 48 hrs. mRNA isolated from triplicates of 3 kidneys with a total of 9 kidneys per condition. All error bars indicate SEM. (**E**) E12.5 *TCF/Lef::H2B-GFP* kidneys cultured for 48 hrs. (**F**) Isolated mesenchyme cultured

without the ureteric bud. Ub- ureteric bud, ec-ectopic nephron, n-nephron. Culture conditions and labelling are as indicated in figures.

Figure S4 – Relating to Figure 3. Dual inhibition of PI3K and activation of β-cateninTcf/Lef signalling in nephron progenitor cells.

(**A-B**) Immunofluorescent stains in regions of kidneys and ENPs from E12.5 kidneys cultured for 24 hrs. Culture conditions and labelling are as indicated in figures.

Supplementary Movie legends

Movie 1 – relating to Figure 1. E11.5 *Six2CreGFP;tdRFP* kidneys cultured for 69 hrs in control conditions or 20 µM LY294002.

Time-lapse movie showing E11.5 *Six2CreGFP;tdRFP* kidneys cultured for 69 hrs in the conditions specified in the movie. Images of brightfield, GFP, and RFP channels were captured every 20 minutes. Times and scales are as indicated in the movie.

Movie 2 – relating to Figure 3. E12.5 *Tcf/Lef::H2B-GFP* kidneys cultured for 49 hrs in control conditions or 20 µM LY294002.

Time-lapse movie showing E12.5 *TCF/Lef::H2B-GFP* kidneys cultured for 49 hrs in the conditions specified in the movie. Images of bright-field and GFP channels were captured every 20 minutes. Red squares indicate magnified single-channel inserts which show the growth of single UBTs and the surrounding nephron progenitor cells. Times and scales are as indicated in the movie.

Movie 3 – relating to Figure 4. E12.5 *Tcf/Lef::H2B-GFP* kidneys cultured for 48 hrs in specified conditions.

Time-lapse movie showing 6 E12.5 *Six2CreGFP;tdRFP* kidneys cultured for 48 hrs in the conditions specified in the movies. Images of brightfield and GFP channels were captured every 20 minutes. Red square indicates magnified single-channel insert which shows the growth of single UBTs and the surrounding nephron progenitor cells from each kidney. Times and scales are as indicated in the movie.

Supplemental Table

Table 1 – relating to all figures. Inhibitors, Concentrations, References

**Inhibitor**	**Target**	**Vehicle**	**Concentrations**	**Source**	**Cat. No.**	**Reference**
LY294002	PI3K	DMSO	20 - 40 µM	TOCRIS	1130	(Vlahos et al., 1994)
GDC-0941	PI3K	DMSO	3- 4.5 µM	Selleckchem	S1065	(Folkes et al., 2008; Workman et al., 2010)
CHIR99021	GSK3β	DMSO	0.75- 1.5 µM	University of Dundee	-	(Meek et al., 2013; Ring et al., 2003)
LDN-193189	BMPR	DMSO	4 µM	STEMGENT	04-0074	(Cuny et al., 2008; Vogt et al., 2011)
endo-IWR1	Tankyrase	DMSO	2 µM	TOCRIS	3532	(Chen et al., 2009; Karner et al., 2010)

Supplemental Experimental Procedures with extended details

Organ culture and time-lapse E12.5 kidneys were used throughout the work unless it was specified otherwise in the text. All organ cultures were performed at 37 °C with 5% CO_2 on 0.4 µm PET Transwell membranes (Corning). The standard culture media contained: DMEM (SIGMA), 10% FCS, and 1% Pen/Strep. For all data shown, a minimum of 3 kidneys were used to confirm general antibody stains in each condition and for samples where measurements were made at least 6 kidneys were used per condition, as specified in individual sections below. Precautions were made to control for antibody variations, see section below: Quantitative image measurements.

To collect E11.5 mesenchyme, whole kidneys were gently trypsinized (2 min at RT), followed by trypsin neutralisation with full culture media. The mesenchyme was then peeled away from the ureteric bud and set up in a trans-filter set-up together with dorsal spinal-cord isolated from the same embryos as the kidneys were dissected from. This technique has previously been described in for example (Davies, 1994; Davies and Garrod, 1995). Three isolated mesenchymes were grouped and placed for each induction with spinal cord all inductions performed in triplicates.

FACS analyses for cell analyses and RNA isolation E12.5 *Six2*$^{+/GCiP}$; *Rosa26t*^{dRFP} kidneys that had been cultured for 24 hrs were trypsinized (2 min at RT), neutralised in 150 µL 1xPBS with 10% FCS, left for 5 min, dissociated into single cells by pipetting, and passed through a cell-strainer. The cells were sorted for GFP and RFP using a FACSAriaIIIu (BD) into RLT buffer and used for mRNA isolation, cDNA synthesis, and gene expression analyses. Cells from 3 kidneys were grouped to form each mRNA isolate replicate. Experiments were performed in triplicate with 9 kidneys per treatment. P-values relating to changes in cell number due to treatments as stated on Graphs in Figure S2C. P-values relating to changes in gene expression as stated on Graphs in Figure 2A-C. Student's t-tests were used to compare conditions.

Primary and Secondary Antibodies Anti-CDH1 (BD Transduction Laboratories, 610182); Anti-JAG1 (R&D Systems, AF599), Anti-WT1 (Santa Cruz, sc-192), Anti-PAX2 (Covance, PRB-276P); Anti-SIX2 (LSBio, LS310189); Anti-phospho AKT (Cell Signalling, 4060); Anti-phospho mTORC1 (Cell Signalling, 5536); anti-LEF1 (Cell Signalling); Anti-β-catenin (BD Transduction Laboratories, 610154); Anti-ZO1 (DSHB R26.4C); Anti-β-laminin (SIGMA, L9393); Hoechst 33342 (Invitrogen, H3570).

Secondary antibodies against mouse, rabbit, goat, and rat IgG were purchased from Invitrogen. Anti-mouse IgG 488, A21202; Anti-rabbit IgG 594, A21207; Anti-goat IgG 488, A11055; Anti-goat IgG 594, A11058; Anti-mouse IgG 647, A31571; Anti-rabbit IgG 488, A21202; Anti-goat IgG 350, A21081; Anti-rabbit IgG 647, A31573; Anti-goat IgG 647, A21447.

NucView 488 Caspase-3 (Biotium, 30029-T-BT) was used to detect apoptosis in response to PI3K inhibition. Live cultures were incubated in 5 µM NucView for 60 min prior to fixation in 4% PFA and immunostaining for additional markers.

Microscopy The time-lapse microscopy was performed on a Nikon TiE (Perfect Focus System) with NIS-Elements 4.0. The imaging was performed using 4X or 10X objectives and a CoolSnap HQ2 CCD camera (Photometrics). The CoolSnap HQ2 CCD camera was used at a range of 0-16383. The microscope stage was enclosed within a humidified chamber at 37 °C with 5% CO_2. Images were captured every 20 minutes of relevant channels (brightfield/RFP/GFP). A combined exposure time for all channels was limited to 2s to prevent phototoxicity.

Confocal microscopy was carried out on a Nikon A1R using 10X-63X objectives, or a N-STORM/A1 super resolution microscope using 10X-63X objectives, or on a Zeiss LSM710 with 10X-63X objectives. Image stitching was carried out at 10X-20X to capture whole kidneys when required. The Nikon microscopes were used with Nikon NIS-Elements 4.0 and the Zeiss microscope with ZEN Black 2012. For image processing Fiji (http://fiji.sc/), ImageJ (http://rsb.info.nih.gov/ij/), and Adobe Photosphop CS5 were used. For image analysis Fiji and ImageJ were used. Time-lapse movies were presented using ImageJ and Fiji. Movies were generated in ZEN Blue 2012. Brightness and contrast levels were adjusted only after any quantification was performed and for presentational purposes only.

Quantitative image measurements When measurements were made between control and experimental samples, kidneys were stained in the same tube and in the same solution. Images were captured using the same settings and care was taken to ensure image intensity ranges were appropriate for comparisons to be made.

Kidney growth in response to LY294002: The area of each explants kidney was measured at the widest point of each kidney. The free-hand drawing tool in Fiji was used to trace the periphery of the kidneys. The portion of the ureteric bud stalk that projected outside the

kidney was not included in the measurements. Student's t-tests were used to compare conditions and p-values are displayed on graph in Figure 1B. 9 and 8 kidneys used for control and Ly294002 conditions, respectively.

Measurements of nephron size in response to LY294002: The width of nephron tubules were measured at the widest point of each tubule within the z-plane captured. Student's t-tests were used to compare conditions and p-values are displayed on graph in Figure 1C. 9 and 8 kidneys used for control and Ly294002 conditions, respectively.

Measurements of nephron number, tip number, and Jag1^{+}area: Kidneys stained for Wt1, Jag1, and Cdh1, were scanned on a confocal. Nephrons and tips were counted by eye on maximum intensity z-projections. JAG1 areas were measured by thresholding the images and using the Particle Analyser to measure individual nephrons. Student's t-tests were used to compare conditions. P-values are shown on Graphs in Figure SJ. A total of 12 kidneys were used, 6 for each condition.

List of TaqMan Primers and Probes (Target/Ensemble ID/Primer 1/Primer 2/UPL probe)
Lhx1/ENSMUSG00000018698/aatgcaacctgaccgagaag/cgcatttggtaccgaaacat/probe7,
Bmp2/ENSMUSG00000027358/cggactgcggtctcctaa/ggggaagcagcaacactaga/probe49,
Jag1/ENSMUSG00000027276/gaggcgtcctctgaaaaaca/acccaagccactgttaagaca/probe6,
Wnt4/ENSMUSG00000036856/ctggactccctccctgtctt/atgcccttgtcactgcaaa/probe62,
Osr1/ENSMUSG00000048387/agaagcgtcagaagtctagttcg/ggaaccgcaatgatttcaa/probe50,
Six2/ENSMUSG00000024134/caagtcagcaactggttcaaga/actgccattgagcgagga/probe5,
Cited1/ENSMUSG00000051159/gaggcctgcacttgatgtc/tggagtaggccagagagttca/probe12,
Lef1/ENSMUSG00000027985/tggttaacgagtccgaaatca/agaggacggggcttgtct/probe20,
Axin2/ENSMUSG00000000142/gagagtgagcggcagagc/cggctgactcgttctcct/probe96,
Irx2/ENSMUSG00000001504/gaagcaaggaggagagttcaga/gtgagcgagtcgacgtgtag/probe98,
HeyL/ENSMUSG00000032744/ctgaattgcgacgattggt/gcaagacctcagctttctcc/probe25,
Lgr5/ENSMUST00000020350.8/cttcactcggtgcagtgct/gatcagccagctaccaaatagg/probe60.

Supplemental References

Chen, B., Dodge, M.E., Tang, W., Lu, J., Ma, Z., Fan, C.W., Wei, S., Hao, W., Kilgore, J., Williams, N.S., *et al.* (2009). Small molecule-mediated disruption of Wnt-dependent signaling in tissue regeneration and cancer. Nature chemical biology *5*, 100-107.

Cuny, G.D., Yu, P.B., Laha, J.K., Xing, X., Liu, J.F., Lai, C.S., Deng, D.Y., Sachidanandan, C., Bloch, K.D., and Peterson, R.T. (2008). Structure-activity relationship study of bone morphogenetic protein (BMP) signaling inhibitors. Bioorganic & medicinal chemistry letters *18*, 4388-4392.

Davies, J. (1994). Control of Calbindin-D-28k Expression in Developing Mouse Kidney. Dev Dynam *199*, 45-51.

Davies, J.A., and Garrod, D.R. (1995). Induction of early stages of kidney tubule differentiation by lithium ions. Dev Biol *167*, 50-60.

Folkes, A.J., Ahmadi, K., Alderton, W.K., Alix, S., Baker, S.J., Box, G., Chuckowree, I.S., Clarke, P.A., Depledge, P., Eccles, S.A., *et al.* (2008). The identification of 2-(1H-indazol-4-yl)-6-(4-methanesulfonyl-piperazin-1-ylmethyl)-4-morpholin-4-yl-t hieno[3,2-d]pyrimidine (GDC-0941) as a potent, selective, orally bioavailable inhibitor of class I PI3 kinase for the treatment of cancer. J Med Chem *51*, 5522-5532.

Karner, C.M., Merkel, C.E., Dodge, M., Ma, Z., Lu, J., Chen, C., Lum, L., and Carroll, T.J. (2010). Tankyrase is necessary for canonical Wnt signaling during kidney development. Dev Dyn *239*, 2014-2023.

Meek, S., Wei, J., Sutherland, L., Nilges, B., Buehr, M., Tomlinson, S.R., Thomson, A.J., and Burdon, T. (2013). Tuning of beta-catenin activity is required to stabilize self-renewal of rat embryonic stem cells. Stem cells *31*, 2104-2115.

Ring, D.B., Johnson, K.W., Henriksen, E.J., Nuss, J.M., Goff, D., Kinnick, T.R., Ma, S.T., Reeder, J.W., Samuels, I., Slabiak, T., *et al.* (2003). Selective glycogen synthase kinase 3 inhibitors potentiate insulin activation of glucose transport and utilization in vitro and in vivo. Diabetes *52*, 588-595.

Vlahos, C.J., Matter, W.F., Hui, K.Y., and Brown, R.F. (1994). A specific inhibitor of phosphatidylinositol 3-kinase, 2-(4-morpholinyl)-8-phenyl-4H-1-benzopyran-4-one (LY294002). J Biol Chem *269*, 5241-5248.

Vogt, J., Traynor, R., and Sapkota, G.P. (2011). The specificities of small molecule inhibitors of the TGFss and BMP pathways. Cell Signal *23*, 1831-1842.

Workman, P., Clarke, P.A., Raynaud, F.I., and van Montfort, R.L. (2010). Drugging the PI3 kinome: from chemical tools to drugs in the clinic. Cancer Res *70*, 2146-2157.
